# Production of Monoclonal Antibodies to Pathologic β-sheet Oligomeric Conformers in Neurodegenerative Diseases

**DOI:** 10.1038/s41598-017-10393-z

**Published:** 2017-08-29

**Authors:** Fernando Goñi, Mitchell Martá-Ariza, Daniel Peyser, Krystal Herline, Thomas Wisniewski

**Affiliations:** 1 Center for Cognitive Neurology and Department of Neurology, New York University School of Medicine, New York, New York USA; 2 Department of Pathology, New York University School of Medicine, New York, New York USA; 3 Department of Psychiatry, New York University School of Medicine, New York, New York USA

## Abstract

We describe a novel approach to produce conformational monoclonal antibodies selected to specifically react with the β-sheet secondary structure of pathological oligomeric conformers, characteristic of many neurodegenerative diseases. Contrary to past and current efforts, we utilize a mammalian non-self-antigen as an immunogen. The small, non-self peptide selected was covalently polymerized with glutaraldehyde until it reached a high β-sheet secondary structure content, and species between 10–100kDa that are immunogenic, stable and soluble (p13Bri). Inoculation of p13Bri in mice elicited antibodies to the peptide and the β-sheet secondary structure conformation. Hybridomas were produced and clones selected for their reactivity with at least two different oligomeric conformers from Alzheimer’s, Parkinson and/or Prion diseases. The resulting conformational monoclonals are able to detect pathological oligomeric forms in different human neurodegenerative diseases by ELISA, immunohistochemistry and immunoblots. This technological approach may be useful to develop tools for detection, monitoring and treatment of multiple misfolding disorders.

## Introduction

**Figure 1:**
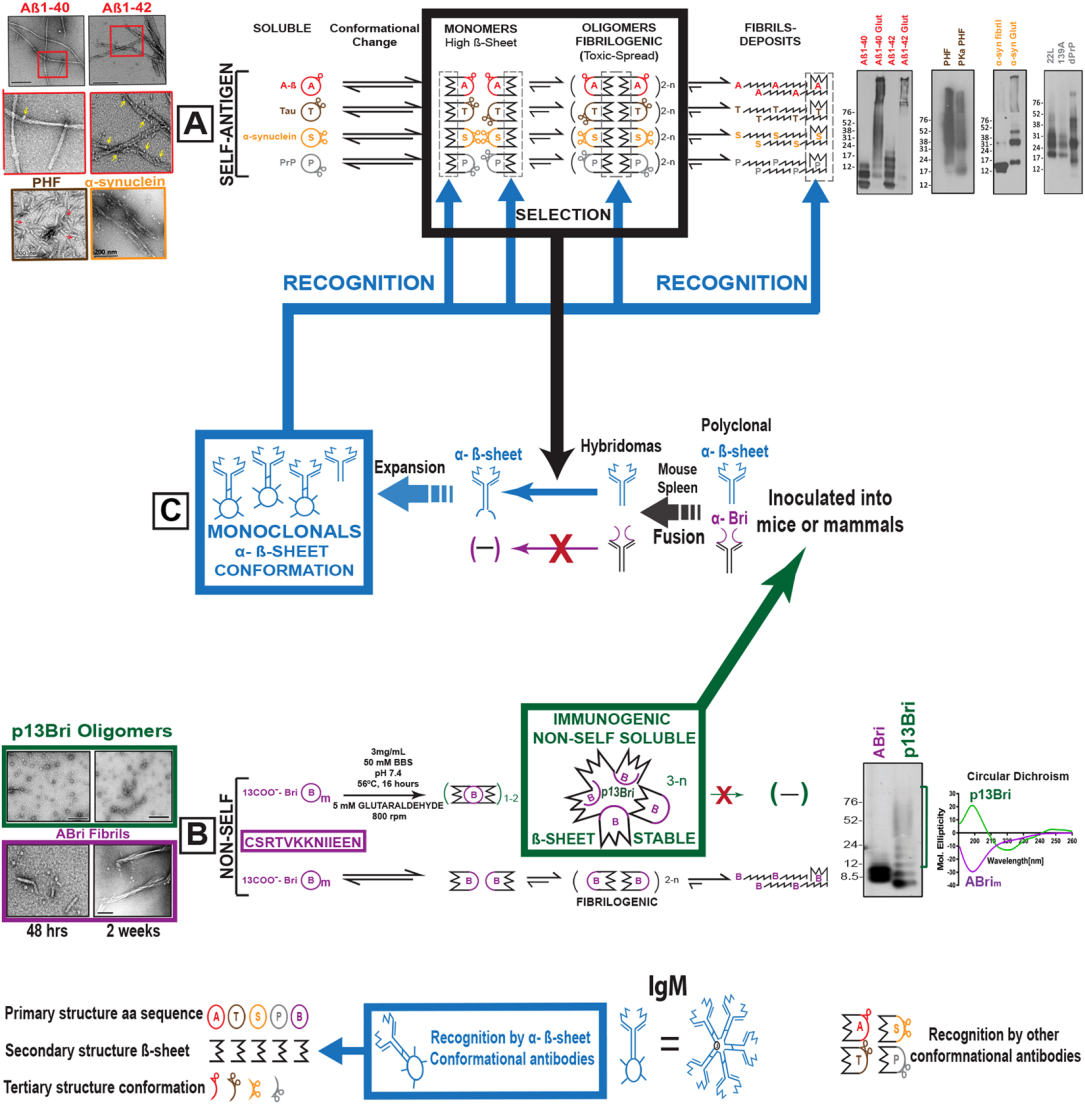
Production of anti ß-sheet secondary structure conformational monoclonal antibodies with specificity to oligomeric toxic conformers present in neurodegenerative diseases (NDD). (**A**) Color coded pathways to oligomeric forms and fibrillar deposits of self-antigenic protein/peptides associated with pathology on most common NDD: Aß (red) and tau (brown) for Alzheimer’s Disease; α-synuclein (orange) for Lewy Body diseases, and PrP (grey) for prionoses. Black shape represents common to all ß-sheet secondary structure acquired during pathological conformational change. Electron microscopy (EM) of oligomers and fibrils on the left and immunoblots of oligomeric forms detected by specific antibodies on the right, all color coded (also in Figs 2, 3 and 4). (**B**) One letter code of the 13 amino acids sequence of the non-self ABri peptide (purple boxed). Bottom pathway shows the normal conversion of ABri peptides to oligomers and fibrils (purple). Top pathway shows the controlled polymerized reaction with glutaraldehyde (see methods) leading to p13Bri the immunogenic, non-self, soluble and stable ß-sheet oligomers of 10–100 kDa molecular weight (from purple to green frame). Black shapes represent common to all ß-sheet structure. On the left, EM of the oligomeric p13Bri (green frame) and the oligomer/fibrils of the aged ABri peptide (purple frame). On the right Immunoblot with rabbit polyclonal anti-Bri and circular dichroism analysis of both forms (color coded and also in Supplementary Fig. 2). (**C**) The p13Bri (green boxed) inoculated into mice to produce hybridomas (Methods and Table 1); horizontal blue arrows show the selection process of monoclonals by the oligomeric β-sheet conformers of (A) antigens (thick black frame and arrow); framed in blue the selected anti-conformational monoclonal antibodies that recognize ß-sheet secondary structure common to (**A**) and (**B**) pathways. The thick blue arrows from the framed blue antibodies signal possible interactive sites with pathological conformers on NDD (also in Figs 3, 4 and 5).

Most neurodegenerative diseases (NDD) develop when a soluble physiologic peptide or protein changes to a new folding characterized by a dominant β-sheet secondary structure and oligomerizes into pathologic, fibrillogenic conformers, which lead to loss of function and toxicity^1–3^. NDD are among the most common causes of disability and death worldwide, posing a massive medical, social and economic burden, as well as scientific challenges. The most common NDD is Alzheimer’ disease (AD) characterized pathologically by its signature lesions of amyloid β (Aβ) deposits in the form of extracellular plaques and vascular amyloid; as well as, tau protein aggregates in the form of intracellular paired helical filaments (PHF) in neurofibrillary tangles (NFT)^4, 5^. However, precursor soluble oligomeric forms of Aβ and tau, which may spread via a “prion-like” mechanism, are thought to be the chief mediators of toxicity in AD^2, 3, 6–10^. Other NDD include Parkinson’s disease (PD), Lewy body dementia (LBD) and prion diseases where the pathogenesis is associated with a similar aggregation/oligomerization process of α-synuclein and PrP^Res^, respectively^2, 11–13^. The change in conformation to the oligomeric or fibrillogenic misfolded conformers opens the window for immunological recognition; hence, immunotherapy, either active or passive, has been a valid therapeutic option for NDD^14, 15^. Initial attempts at active vaccination in AD failed in part due to autoimmune toxicity from the use of self-immunogens, such as aggregated Aβ^16^. Clinical trials of passive immunization have also produced disappointing results related to the targeting of both physiological and pathological forms of Aβ, without specific targeting of the most toxic species^14, 15, 17, 18^. It is now recognized that the soluble toxic oligomeric forms of pathologic proteins or peptides might be more efficient immunologic targets in both immunotherapeutical approaches (Fig. 1). This approach has led to the production of a few anti-conformation monoclonal antibodies and new formulation vaccines, as was previously reviewed^14, 15^.

Two potential problems must still be addressed for possible therapeutic success of interference with toxic oligomeric structures. The first is using primary structure self-antigens for oligomeric tertiary structure immunogens for active immunization or for the production and selection of anti-conformation monoclonal antibodies, with the latent possibility of autoimmune toxicity. The second is the restrictive specificity of the immunogen to a single or limited number of pathological conformers^18^ (Fig. 1).

To overcome these problems we developed a methodology to produce conformational anti-secondary structure β-sheet monoclonal antibodies. The β-sheet secondary structure of proteins can be derived from many different primary sequences, but generally is dominant in the production of any pathologic misfolded proteins or peptides. As an immunogen we used a small 13 amino acids peptide of the carboxyl terminus of the very rare British amyloidosis (ABri), which is derived from an intronic DNA sequence expressed by a missense mutation and has no sequence homology to any other mammalian protein^14, 19–21^. The peptide was polymerized by an extensive glutaraldehyde reaction to form immunogenic, covalently bound 10–100kDa soluble and stable oligomers with high β-sheet secondary structure content (p13Bri)^21, 22^. Inoculated in mice with a suitable adjuvant p13Bri produced an array of antibodies to the non-self motif and the β-sheet secondary structure. Hybridomas were produced and monoclonals selected by the novel approach of specifically using as selector compounds, oligomeric conformers from different NDD with the only commonality being the shared β-sheet secondary structure (Fig. 1). These new monoclonals to β-sheet conformation in oligomers may more effectively detect, monitor and treat NDD in humans and other susceptible animals.

## Results

The ABri peptide selected as an immunogen is only 13 amino acids long with no sequence homology to any known mammalian protein. It can adopt a ß-sheet secondary structure and by aggregation form protofibrils evolving into an amyloid fibrillar form (Fig. 1B, purple pathway and Supplementary Fig. 1). To avoid fibril formation that previously complicated the immune response in humans we selected glutaraldehyde for cross-linking polymerization. The ABri has two preferential Lysyl residues at positions 6 and 7 amenable to the covalent linkage of more than one unit through a glutaraldehyde bridge^21, 22^. However, the free NH_2_ group from the amino-terminus of a very small peptide, or the two adjacent Lysyl residues could be prone to form a Schiff base with the glutaraldehyde preventing further association^23, 24^. Thus, we selected a mild alkaline pH for the reaction to stabilize the Lysines net charges and maximize separation by charge repulsion, a high temperature and high rpm shaking to avoid stabilizing a blocked monomeric structure during the chemical reaction, a buffer with no phosphate or Tris groups that could interfere with the progression of oligomerization, and a glutaraldehyde concentration to equalize the ratio of long self-polymerized glutaraldehyde chains versus the joining of two or more ABri peptides^21, 22, 24^. Nevertheless, some monomers, dimers and trimers were formed as detected in gels by protein stain or specific antisera (Fig. 1B, green pathway, EM, CD and blot and Supplementary Fig. 1). These lower molecular weight forms still consistent of a predominant β-sheet secondary structure, but never aggregate into fibrils. The rest of the covalently linked oligomers distributed between 10 and 100kDa, remained stable for very long periods of time, never forming potentially cross-seeding fibrils. This resulting p13Bri immunogen is composed from many intermediate size covalently linked oligomers with a high number of repetitions of the small 13mer motif in a predominant ß-sheet secondary structure (Fig. 1 and Supplementary Figure 1).

The previously published p13Bri vaccine inoculated with Aluminum Hydroxide (Alum) as an adjuvant produced a mild polyclonal response to pathologic oligomeric forms present in three mouse models of AD; i.e.,Tg APP/PS1 (with mainly amyloid plaques), Tg SwDI (with extensive vascular amyloid) and 3xTg APP/PS1 P301L (with combined Aβ and tau pathologies)^14, 21, 22^. In all three models AD pathology was greatly reduced and cognitive rescue was achieved by early vaccination^14, 21, 22^. To reproduce the successful immune response, increasing the sustainable antibody concentration for monoclonal production purposes and avoiding possible interference from the transgenes of the AD mouse models, we inoculated 5 wild type CD-1 mice with a modified protocol, including some with a RiBi-like Sigma adjuvant to enhance the antibody immune response (Supplementary Table 1 and in Materials & Methods).

**Figure 2:**
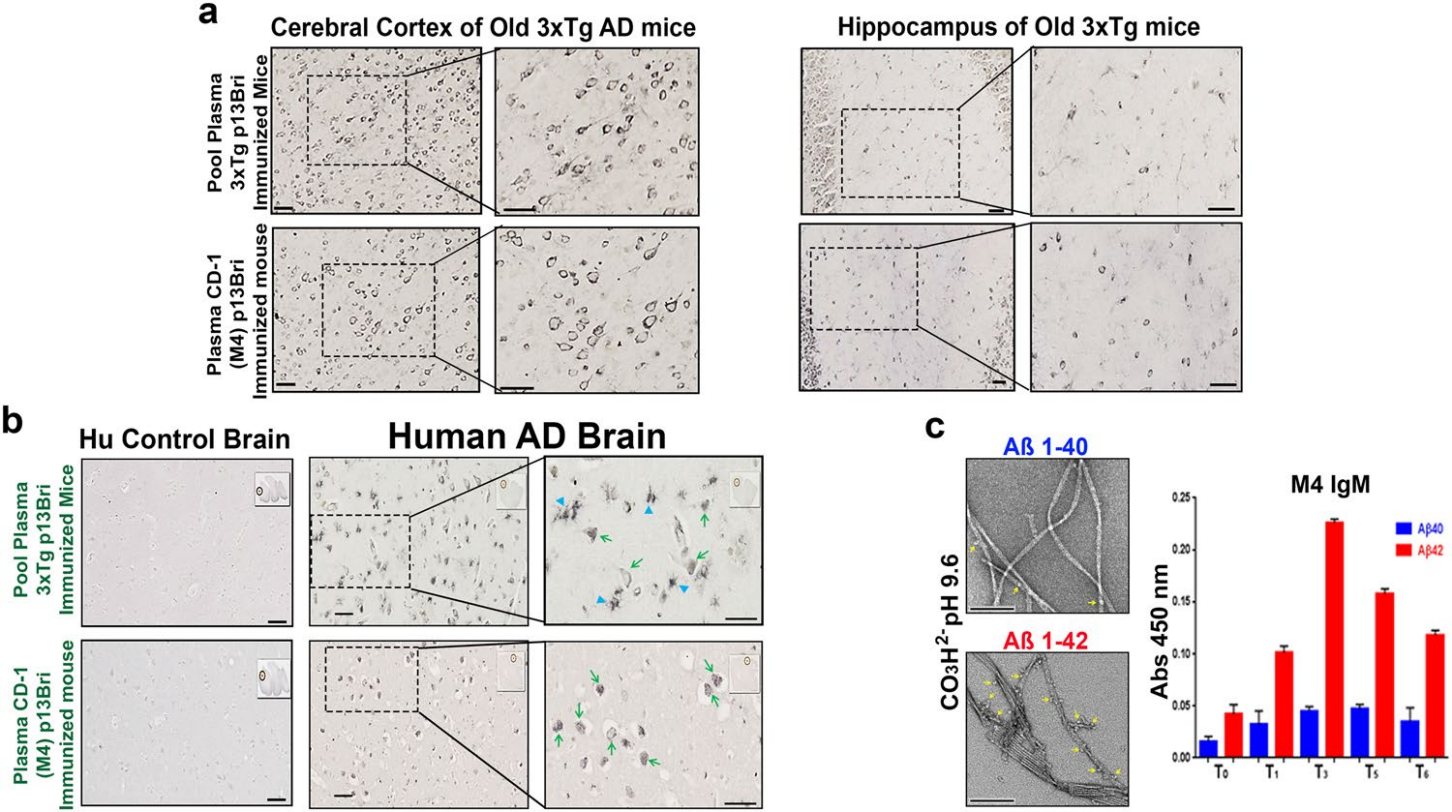
Immunochemistry of plasma from p13Bri immunized 3xTg AD mice and CD-1 M4 mouse on old 3xTg AD mouse model brains, human AD and control brains; and immunoreactivity of M4 plasma on Aβ40/42 ELISA. (**a**) Similarities of plasma reactivity from 3xTg mice successfully immunized with p13Bri (top)22 and CD-1 M4 mouse (bottom) on cerebral cortex and Hippocampus of old 3xTg AD mice with amyloid and tau pathology. Right panels show higher magnification of the boxed areas. All scale bars represent 50 µm. (**b**) Same plasma comparison as in a) but in the cortex of human AD brains. Left panels show negative reactivity on human control brains. Right panels show magnification of the boxed areas. Arrowheads show glial-like cells; arrows show cytoplasmic punctuated staining in neurons. Scale bars represent 50 µm. (**c**) Electron Microscopy (EM) images of Aβ1–40 and Aβ1–42 peptides on 50mM bicarbonate pH 9.6 used to coat ELISA plates. Yellow arrows show oligomeric forms decorating amyloid fibrils in both cases. Scale bars represent 200 µm. Right panel shows ELISA differential IgM reactivity to Aβ1–40 and Aβ1–42 from plasma of the M4 CD-1 mouse at different bleeding times as per Table 1.

We analyzed the elicited polyclonal antibody response by ELISA with differential Aβ1–40 and Aβ1–42 coats known to develop β-sheet aggregation^21, 22^. Each Aβ peptide was dissolved in bicarbonate buffer pH 9.6 and left at RT to age; the Aβ1–40 for a few hours before coating and the Aβ1–42 for at least two days before coating; enough in both cases to establish aggregation and fibrillization. EM analysis of both Aβ peptides after aging, documented fibril formation; however, because of the high pH, a significant number of stable oligomeric forms surrounded the fibrils of Aβ1–42 at a higher concentration per area than with the Aβ1–40 fibrils (Fig. 2c). The polyclonal IgM and IgG response obtained in CD-1 animals was similar to the one reported in the 3xTg inoculated animals^22^, and could distinguish the misfolded peptides and the differential concentration of oligomeric forms in both of them, with a much higher titer against Aβ1–42 versus Aβ1–40 (Fig. 2c and Supplementary Figure 2). The control to assess for equal coating of both Aβ peptides was determined with two commercial IgG anti-Aβ primary structure monoclonal antibodies (mAbs) 4G8 and 6E10, that have similar reactivity for both Aβ1–40 and Aβ1–42 (Supplementary Figure 2c), demonstrating the differential data obtained from p13Bri immunized animals depended on the recognition of misfolded oligomeric forms (present at a higher concentration in the Aβ1–42 preparation) rather than an unspecific cross-reaction to the primary structure of the peptides used for plate coating.

By immunohistochemistry the plasma of the CD-1 inoculated animals as well as the previously reported pooled plasma from successfully vaccinated animals recognized similar neuronal cytoplasmic and extracellular material in the cerebral cortex and hippocampus of untreated old 3xTg mice with extensive amyloid-β and tau pathologies (Fig. 2a)^21, 22^. The same plasma samples were used to immunolabel the temporal cortex of human AD brains. Both the plasma of previously vaccinated animals^21, 22^ and the plasma from p13Bri immunized CD-1 mice recognized comparable intracellular and extracellular pathologic features in human AD sections but showed no significant immunolabeling in the cerebral cortex of human control brains with no AD pathology (Fig. 2b). Both IgM and IgG antibodies from the CD-1 animals co-localized with the same pathological structures in human AD brains (Supplementary Figure 3).

To test the feasibility of producing hybridomas with specific anti-β-sheet secondary structure, we first selected the CD-1 animal M4 that had the best IgM and IgG polyclonal response to β-sheet oligomers (Fig. 2 and Supplementary Figure 3). The protocol of the M4 p13Bri inoculations and fusion used for subsequent hybridoma production is shown on Table 1 and is described in the Materials & Methods (M&M).

**Table 1:** Protocol of Immunization of mouse M4 with p13Bri for the Production of Conformational Antibodies Cross-reacting to Oligomeric Forms of Proteins and Peptides found in Neurodegenerative Diseases.

Date of Inoculation (in days)	p13Bri Antigen Amount (μg/animal)	Antigen to Adjuvant Ratio*	Route of Inoculation	Identification of Bleed	Date of Bleed (in days)
—	NONE	n/a	NONE	Pre-Immune T0	−7
0	50	4:1	s.c	—	—
14	50	4:1	s.c	—	—
—	—	—	—	T1	21
28	20	9:1	s.c	—	—
—	—	—	—	T2	35
49	20	9:1	s.c	—	—
—	—	—	—	T3	56
69	20	9:1	s.c	—	—
—	—	—	—	T4	76
91	20	9:1	s.c	—	—
—	—	—	—	T5	98
119	20	9:1	s.c	—	—
—	—	—	—	T6	126
165	**10**	**no adjuvant**	**i.v**	—	—
169	Splenocytes fused to SP2/0-IL6**	—	—	**TTB M4**	169

*Sigma adjuvant system SC: Subcutaneous; IV: Intravenous; **Fusion Partner; TTB: Terminal bleeding.

After fusion of the M4 mouse spleen cells to the SP2/0-IL6 partner cells, the fused cells and the viable hybridomas were selected by incubation and serial dilution as per Table 1 and the Monoclonal Production method described in the M&M.

**Figure 3:**
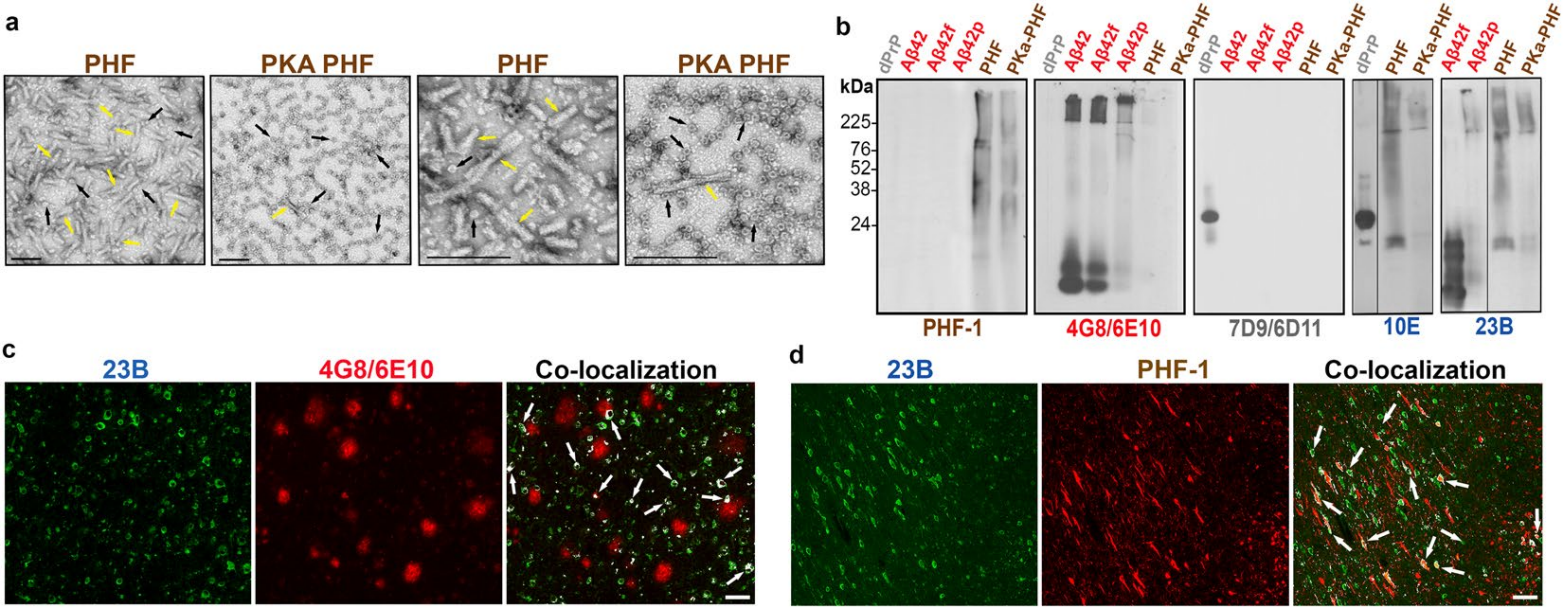
Electron Microscopy of paired helical filaments (PHF) and PKA treated PHF, and comparative detection of specific NDD conformers. (**a**) EM of purified PHF from a human AD brain and protein kinase A treated PHF. Yellow arrows show fibrils and black arrows show oligomers in different aggregation clusters. Left two panels show overall difference of fibrils and oligomers in both samples and right two panels are higher magnification to show the oligomers associated to fibrils or in independent clusters. All scale bars are 50 µm. (**b**) Immunoblots of recombinant deer PrP (grey dPrP); Aβ1–42 freshly dissolved, fibrilized or polymerized (red Aβ42, Aβ42f and Aβ42p respectively), and PHF and PKA PHF (brown). Individual specificity of commercial antibodies PHF-1 for hyperphosphorylated tau; 4G8 and 6E10 for Aβ peptides and 7D9 and 6D11 for PrP protein is compared to the cross-reactivity of hybridomas 10E and 23B (blue) reactive to more than one conformer and oligomeric forms (the uncropped blots showing 10E and 23B reactivity are shown in Supplemental Figure 6). (**c** and **d**) Co-localization of hybridoma 23B with either 4G8/6E10 or PHF-1 antibodies on human AD brain tissue. Scale bar represents 100 µm.

**Figure 4:**
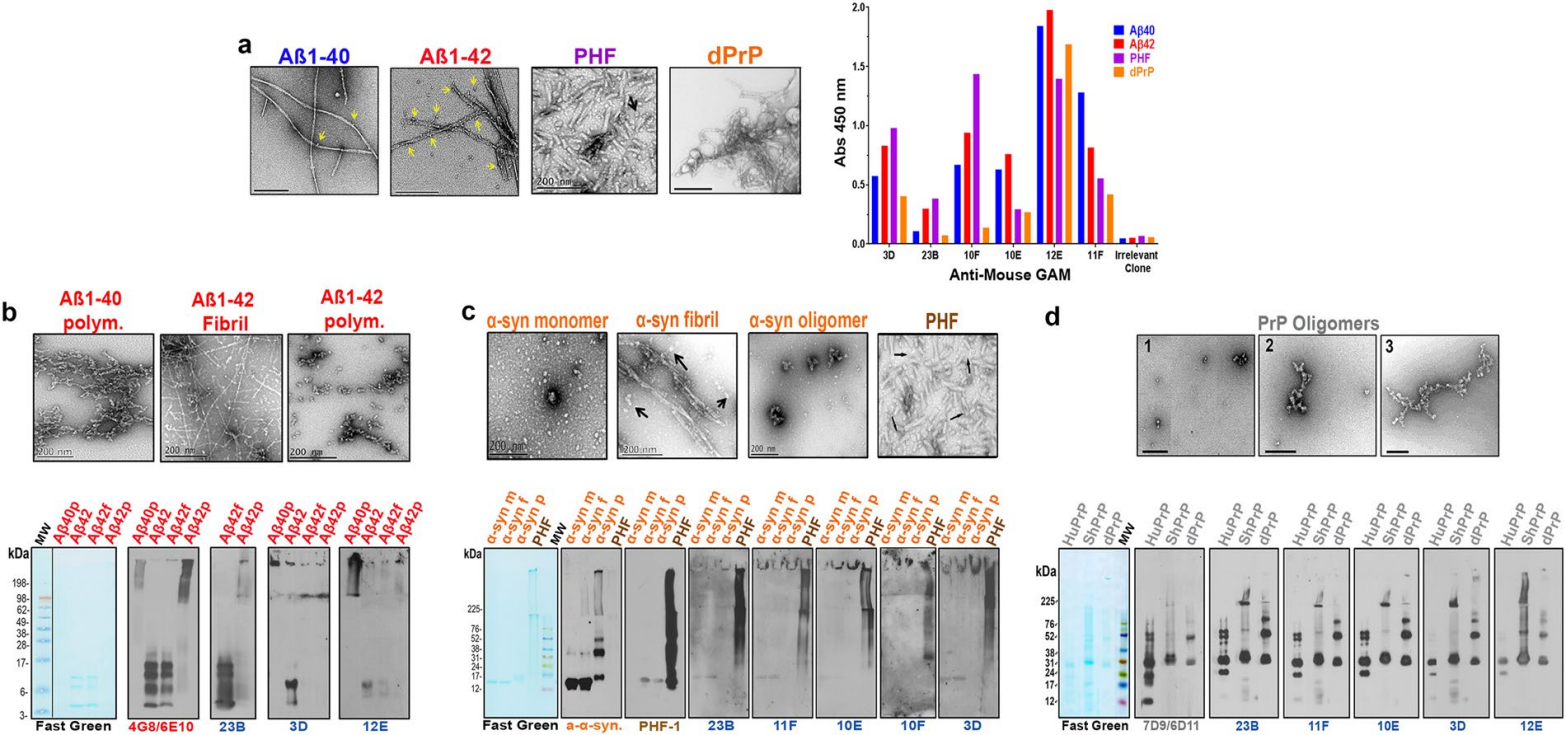
Electron Microscopy of fibrillar and oligomeric forms of misfolded protein/peptides from NDDs and the recognition in ELISA and immunoblots by the original hybridomas 3D, 23B, 10E, 11F, 10F and 12E. (**a**) EM of Aβ1–40, Aβ1–42, PHF purified from a human AD brain, and oligomerized PrP; yellow arrows show oligomeric forms decorating amyloid fibrils. The right panel shows the ELISA reactivity of original positive or irrelevant clones to four neuroconformers. (**b**) Top, EM of Aβ1–40 and Aβ1–42 polymerized with glutaraldehyde and Aβ1–42 fibrillized, and the corresponding immunoblots (bottom): Molecular weight marker (MW) followed by Aβ1–40 polymerized (Aβ40p), Aβ1–42 freshly dissolved (Aβ42), Aβ1–42 fibrillized (Aβ42f) and Aβ1–42 polymerized (Aβ42p). The left panel shows reversible Fast Green (FG) protein stain to assess comparable protein load. The next panel shows the reactivity with commercial IgG monoclonals 4G8 and 6E10 (labeled in red) specific for Aβ peptides sequence. The three right panels show positive clones (labeled in blue) with differential reactivity to oligomeric forms of Aβ. (**c**) Top, EM of α-synuclein monomer, fibrillized on PBS or oligomerized with glutaraldehyde, and PHF. Bottom panels corresponding immunoblots lanes: α-synuclein monomer (α-syn m), α-synuclein fibrillized (α-syn f), α-synuclein oligomerized (α-syn p) and PHF. Left panel FG, next panel commercial anti-α-synuclein antibody (labeled in orange); third from left commercial PHF-1 (labeled brown) and to the right five original clones (blue). (**d**) Top, EM of different states of aggregation of aged recPrP molecules. Bottom, immunoblots lanes: human (HuPrP), sheep (ShPrP) and deer PrP (dPrP). All PrPs were incubated for at least two days to maximize aggregation. Left panel FG, second panel commercial IgG monoclonals 7D9 and 6D11 (labeled in grey) that recognize middle parts of PrP. Next five panels show the reactivity of 5 original clones (blue) with differential oligomer size detection previously unseen.

The anti-β-sheet secondary structure selection process was novel for monoclonal production and involved the simultaneous detection of reactivity of plated viable hybridomas with four different NDD conformers. The small availability, in each round, of cell supernatant from three days growth of a limited number of hybridoma cells in 96 well plates required a sensitive ELISA differential analysis. The concentration of Aβ oligomers around fibrils of Aβ1–40 and Aβ1–42 samples was described above (Fig. 2c) and required two separate ELISA plates for analysis. Two additional ELISA plates were used: one plated with PHF extracted from a human AD brain and treated to maximize the number of clusters of β-sheet oligomeric conformers associated with the fibrils (Figs 3a and 4a) that have been reported to be associated with β-sheet steric zippers characteristic of toxic oligomerization before they become buried in fibril structures^25, 26^; and another plated with an aged elk recombinant PrP produced in *E. coli*.^27^, which has properties resembling PrP^Res^ with oligomerization and exposed β-sheet motifs, without the extended β-sheet structure characteristic of amyloidogenic and infectious PrP^Sc^ (Figs 3b and 4a,d)^12, 28–30^.

In the first round of selection, more than fifty limited volume supernatants from samples of wells containing a few cells were analyzed at the same time precluding a further dilution for duplicates or for individual plates to assess IgG and IgM classes, separately. A polyclonal anti-mouse GAM, at a suitable dilution, was used to maximize detection. As a positive control, commercial antibodies to each specific sequence conformer on the corresponding plates were used, assuring the homogeneity of the coating. At the same time the anti-mouse GAM was able to recognize IgM or IgG hybridomas producing readings of at least three times over the background as shown by comparison to irrelevant clones (Fig. 4a and Supplementary 5a and b). Cells from all supernatants that were positive with at least two of the four conformational selectors were subcloned as described in M&M. The clones that were positive for at least two conformers and maintained the reactivity for three rounds of subcloning were deemed potential anti-β-sheet monoclonals, separated and expanded (Fig. 1C, blue pathway).

**Figure 5:**
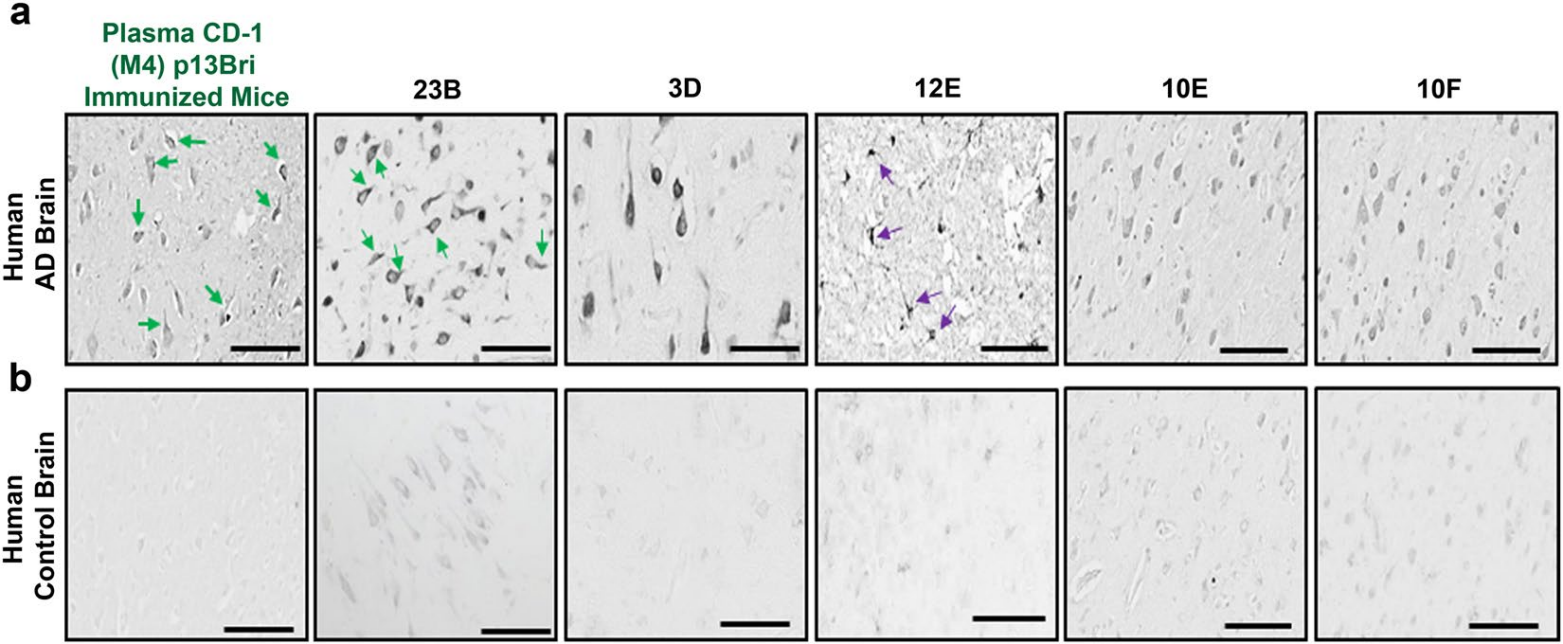
Immunohistochemistry of plasma from p13Bri immunized M4 mouse and five partially purified anti ß-sheet secondary structure conformational monoclonal antibodies on human AD and control brains. (**a**) Immunolabelling of human AD brain cortex. (**b**) Human control brain cortex without pathology. Left panel, reactivity of the plasma from p13Bri immunized M4 mouse (green label), green arrows show cytoplasmic staining that extends to processes. Next five panels ammonium sulfate semi-purified anti ß-sheet secondary structure conformational monoclonals. 23B labels cytoplasm, processes and extracellular material; 3D labels the whole neuronal body; 12E shows preference for glial cells; 10E and 10F show similar lighter staining pattern of neuronal cytoplasm, processes and nucleus. Scale bars represent 50 µm.

Thirty five potential clones were obtained using the above criteria, which could be divided into six families of similarly reacting monoclonals from which the best representatives were 23B; 10E; 3D; 12E; 11F and 10F (Figs 3b; 4 a–d and 5a). All clones that were stable with a sustained production of an anti-β-sheet monoclonal were later shown to be IgM-kappa in pentameric form (Supplementary Figure 4), whereas unexpectedly, no stable IgG producing hybridomas survived the three rounds of selection.

After initial expansion of the potential anti-β-sheet secondary structure clones, enough cell supernatant was available to corroborate the ELISA reactivity in duplicate (results not shown), and was used as a source of primary antibody in specific immunoblots that showed the poly-reactivity to the same antigens that shared only a dominant β-sheet secondary structure, as well as to oligomerized α-synuclein. The immunoblots also showed specific detection of low abundance oligomeric structures from every NDD conformer that were typically detected less well or not at all by the anti-primary structure dependent commercial antisera specific for only one type of protein or peptide (Fig. 3b).

To assure the anti-β-sheet conformation reactivity was due only to IgM monoclonals; all six representative monoclonals were partially purified by ammonium sulfate precipitation (SAS) to remove more than 90% of the BSA and other contaminating proteins. The integrity of the IgMκ pentamer was maintained as well as the antibody specificity (Supplementary Figure 4).

Each one of the representative monoclonals reacted in gels in a specific way with the different NDD oligomeric conformers (Fig. 4b–d), including reactivity to polymerized recombinant α-synuclein aggregated at the top of the gels, that has similarities to the toxic α-synuclein oligomers that are found in PD and LBD (Fig. 4c). Each selected monoclonal shows evidence of cross reactivity, with reactivity to at least two oligomeric conformers with differing primary sequence (Fig. 4b–d).

The SAS semi-purified monoclonals were used to immunolabel the cortex of human AD brains. Each monoclonal differentially labelled extracellular and cytoplasmic material: 23B strongly labels neuronal cytoplasm including processes and the nucleus; 3D labels the whole neuronal cell body; with both labeling some extracellular material. 12E preferentially labelled glial cells. 10E and 10F showed a lighter immunolabeling of all neuronal cytoplasmic components (Fig. 5a). No appreciable labelling was detectable with monoclonals using cortex tissue of human control brains with no NDD pathology (Fig. 5b). All reactivities can be traced as derived from the polyclonal response of the M4 mouse before fusion (Figs 2a,b and 5a), demonstrating the monoclonals were originally elicited by the p13Bri immunization and selected by using the different NDD conformers.

The generated monoclonals will be made available to requesting researchers in keeping with NIH resource sharing guidelines.

## Discussion

We have developed, using a novel methodology, anti-β-sheet secondary structure monoclonal antibodies to a dominant β-sheet structure that is present in pathology associated oligomers of misfolded protein/peptides of different NDD.

We demonstrate that the production of anti-β-sheet monoclonal antibodies to a particular secondary structure present in oligomers of misfolded protein/peptides can be achieved in a sequential manner that involves first production of a stable oligomer preparation using a small non-self peptide polymerized to itself. This polymerized peptide, which we term p13Bri, is derived from only the last 13 amino acids of the carboxyl terminus of the ABri peptide, oligomerized using glutaraldehyde as a cross linker to form a stable population of sequence homogeneous oligomers, as previously described^19, 20^ (Fig. 1, Supplementary Figure 1 and M&M). The carboxyl 13 residue end of ABri lacks any sequence homology to Aβ, tau or any other native human proteins, since it is derived from an intronic transcript^14, 19–21^. The length of this specific sequence allows it to gain a dominant β-sheet secondary structure, but is too short for significant folding to a tertiary structure, which would introduce unwanted competing conformations in the resulting immunogen. Thus, our polymerization process stabilized repeating motifs with only β-sheet secondary structure, increasing the oligomer size so that it would be immunogenic by itself.

Our prior work using 3 different AD transgenic (Tg) mouse models, has shown that active immunization based on this approach produces a therapeutic polyclonal response that reduces all three key neuropathological features of AD, namely amyloid plaques, congophilic amyloid angiopathy (CAA) and tau related pathology, in association with significant cognitive benefits^14, 15, 21, 22, 31^. Amyloid plaques and CAA were shown to be reduced in APP/PS1 (amyloid plaque model) and TgSwDI (CAA Tg model) model mice, respectively, while in 3xTg mice (amyloid plaque and tau pathology model) p13Bri immunization led to reductions of both tau and Aβ pathology^14, 15, 21, 22, 31^. Inoculation of p13Bri with Alum as an adjuvant in these three AD Tg models produced a systemic polyclonal response to pathologic/oligomeric forms of both Aβ and tau, as well as demonstrating cross-specificity to AD, prion disease, and LBD human brain tissue (Fig. 2 and results not shown). These encouraging and unusual results led us to the production of hybridomas from which we could select monoclonal antibodies with potential diagnostic or therapeutic value, by their specific reactivity to β-sheet secondary structures found in unrelated primary sequences of pathologic conformers of diverse NDD.

In comparison to our previous successful active vaccination using p13Bri with Alum as an adjuvant in AD Tg mice, in wild-type mice such as the CD-1, we used a longer and more intense immunization protocol along with a RiBi-like adjuvant (Supplementary Table 1 and M&M) in order to expand the antibody response to the dominant β-sheet secondary structure in oligomers and generate a greater numbers of spleen B-cells with anti-β-sheet receptors; thus, increasing the chance of being able to transform these B-cells into antibody producing stable hybridomas. Thus, the polyclonal response was analyzed by ELISA tests as described in Results with the understanding that detecting a differential in Aβ oligomers concentration per area around fibrils may reflect more accurately the real biochemical dynamic around plaques in AD^3^. Plasma from the p13Bri immunized CD-1 animals after 6 inoculations clearly showed a persistent IgM response to the oligomeric forms of Aß (Fig. 2c and Supplementary Figure 2). These persistent polyclonal IgMs, similar to what we previously reported^21, 22, 32^, were later shown to be consistent and their IgM producing B-cells able to be transformed into stable monoclonals (Figs 3–5 and Supplementary Figure 2 and 5).

In order to assure we could detect and separate only the clones with specificity to a β-sheet secondary structure conformation, we selected monoclonal clones by testing reactivity to a number of different oligomer preparations from various NDD that only share a common β-sheet secondary structure, but no primary sequence homology. Clones were selected that had strong reactivity to at least two distinct β-sheet secondary structure conformations, with a differing primary sequence. The aged or oligomerized Aß peptides used in ELISA and blots (Figs 3 and 4) reflect the known structures of bend parallel or anti-parallel ß-sheet secondary structure, which will convert to oligomers and eventually fibrils^25, 30, 33^. The PHF purified from AD subjects and the PHF digested with proteinase K were specific selectors of the dominant ß-sheet secondary structure associated with tau toxic oligomers (Figs 3 and 4)^10, 25, 26, 30, 34, 35^, while the deer recombinant PrP served as an example of aggregation through ß-sheet dominant motifs similar to that found in oligomeric PrP^Res^ ^25, 30^. In all cases, these new monoclonals recognized novel oligomeric structures that are not evident using conventional anti-primary structure antibodies (Fig. 3). These monoclonals are NDD pathology specific; however, each clone shows preferential binding to different oligomer species (Figs 4 and 5).

Many oligomer specific antibodies have been reported^1, 36–44^. However, none of these mAbs were demonstrated to be specific for a secondary structure antigen and none have been shown to bind both Aβ oligomers, as well as pathological tau (PHF), with the majority being raised to self-antigens. A11 is a rabbit polyclonal antibody raised to Aβ1–40 bound to gold colloid particles^18, 36^. NAB61 was generated using Aβ1–40 crosslinked with peroxynitrile^37^. BAN2401 was raised to protofibrils of Aβ1–42 with the Arctic mutation^38^. NU-1 was raised to amyloid β-derived diffusible ligands (ADDLs) of Aβ1–42^39^. A-887755 was raised to globulomers of Aβ20–42^40, 42^. ACU-193 was raised to aggregated Aβ^45^. B10 is an antibody binding domain selected for stabilizing Aβ protofibrils^46^, while the two IgMs W01 and W02, both raised against Aβ only recognize generic amyloid fibrils and protofibrillar Aβ but not soluble oligomeric forms^44^. The same group, that produced A11, has also produced OC, a polyclonal, which was raised to Aβ1–42 fibrils^47^ and developed the rabbit IgG mAbs 204 and 205 generated using Aβ1–40 coupled to colloidal gold particles, rather than risk the production of unstable mouse monoclonals^41^. Following the rationale of our original p13﻿Bri vaccination^21^, this group also used a random sequence peptide, called 3A, for active immunization and demonstrated that when coupled to colloidal gold particles the induced polyclonal immune response recognized Aβ oligomers on dot blots; however, the conformation of 3A was not directly documented and importantly the generated polyclonal immune response did not recognize tau pathology^48^. Similarly, none of the aforementioned antibodies directly recognize tau oligomers/tau related pathology. Furthermore, the majority of these mAbs have been characterized with unspecific chemical methods such as dot blots. Hence, although they likely have high affinity for certain specific oligomer species, they might also bind to appropriately folded monomers at a lower affinity. In potential therapeutic settings the concentration of physiological monomeric species is much higher, hampering the effectiveness of such antibodies. Furthermore, all of these mAbs were raised and selected by variations of the same Aβ self-antigen, having the potential issue of late autoimmune toxicity. On the other hand, the anti-tau oligomer specific mAbs (TOMA) have also been raised, using the aggregated tau self-antigen, and shown to reduce tau pathology^43^; however, these do not cross-immunoreact with Aβ oligomers.

Due to the novel method by which we have generated our anti-β-sheet conformational mAbs and their poly-reactivity to toxic conformers found in most common NDD, we believe our approach to be innovative and more likely to have therapeutic success in humans than compared to all other existing oligomer targeting mAbs. The potential advantages are: 1) a diminished risk of inducing auto-immune complications since the immunogen used has no sequence homology to any human peptide/protein (except to the protein expressed in the very rare patients with British amyloidosis); 2) selective targeting of the β-sheet secondary structure found in toxic oligomers, thus avoiding interference with the multiple physiological functions of soluble Aβ, tau and α-synuclein; 3) reduced risk of inducing vasogenic edema/encephalitis related to direct clearance of fibrillar Aβ vascular deposits, since mainly oligomeric forms of Aβ and tau are being targeted; 4) concurrently targeting Aβ, tau and α-syn related pathologic conformers, addressing the mixed pathologies found in the majority of NDD patients^49–52^; 5) minimal risk of increasing toxic oligomer species as shown with some vaccination methods^53^; 6) possible therapeutic use in prion diseases with the potential to interfere with the spread of PrP^Res^. No other reported methodologies to produce mAbs to oligomers published thus far, has this unique combination of properties. Hence we believe that our technological approach has the potential to develop tools for the detection, monitoring and treatment of multiple NDD.

## Materials and Methods

### Synthesis and Polymerization of 13-mer Bri Peptide (p13Bri)

The procedure was performed as previously described^21^. Briefly, the 13 residue peptide that corresponds to the carboxyl terminus of ABri (Cys-Ser-Arg-Thr-Val-Lys-Lys-Asn-Ile-Ile-Glu-Glu-Asn)^4^ was synthetized on an ABI 430A peptide synthesizer (AME Bioscience, Chicago, IL) at the Keck peptide synthesis facility at Yale University, CT. Mass spectroscopy of the lyophilized end-product was used to verify the expected molecular weight.

To have a stable oligomeric conformation and make the 13mer Bri peptide immunogenic by itself, the synthetic peptide was subjected to controlled polymerization using the following protocol: The peptide was dissolved at 3 mg/ml, in 100 mM borate-150 mM NaCl (BBS), pH 7.4. Fresh 1% glutaraldehyde in BBS was added to the peptide to a final 5 mM glutaraldehyde concentration and incubated in an Eppendorf block shaker at 800 rpm and 56 °C for 16 hrs^21, 22^. The solution was then quenched with 0.5 M glycine to make the solution 100 mM in glycine. After five minutes the solution was diluted 1:3 with BBS transferred to a dialysis membrane with a MWCO 2000 (Spectra Laboratories, Rockleigh, NJ) and dialyzed extensively against 200 volumes and three changes of 2 mM BBS at 4 °C, aliquoted and lyophilized. To determine the degree of aggregation the original monomeric ABri peptide and polymerized 13mer Bri peptide (p13Bri) were electrophoresed on 12.5% (SDS)-polyacrylamide Tris-tricine gels together with the low range Rainbow™ molecular weight markers (Amersham Biosciences, Piscataway, NJ) under reducing conditions, then transferred onto nitrocellulose membranes and blotted against a specific rabbit polyclonal anti-Bri (kindly provided by Dr. R. Vidal, IUPUI). For circular dichroism the ABri and p13Bri at 0.25 mg/ml in saline were analyzed as previously described^21^. For electron microscopy studies, the original and polymerized Bri peptides were incubated at 1 mg/ml in 50 mM phosphate-150 mM NaCl (PBS) pH 7.4. Within 24 hours, 3 μl of each sample were placed onto carbon coated 400 mesh Cu/Rh grid (Ted Pella Inc., Redding, CA) and stained with 1% uranyl acetate in distilled water (Polysciences, Inc, Warrington, PA). Stained grids were examined under Philips CM-12 electron microscope and photographed with a Gatan (4 k × 2.7 k) digital camera. The EMs were also repeated on the aged samples kept at room temperature (RT) for one, two and four weeks.

#### Immunization of CD-1 Mice

Immunization of the CD-1 mice and the subsequent hybridoma production were performed at the Bi-Institutional Antibody and BioResource Core Facility of Memorial Sloan Kettering Cancer Center, with screening for the presence of anti-β-sheet conformational mAbs performed at New York University. All experimental procedures were approved by the Memorial Sloan Kettering and New York University Institutional Animal Care and Use Committees (MSK protocol 97–03–009 and NYU protocol 130209–03, respectively) and followed NIH standards. The animals were maintained on a 12 h light/dark cycle, and animal care was in accordance with institutional guidelines in facilities approved by the Association for Assessment and Accreditation of Laboratory Animal Care.

In order to optimize the conformational immune response sought in this experiment, the inoculation schedule and inoculum were determined, prepared and provided (by FG and TW) to the Core Facility. The p13Bri peptide was dissolved in sterile saline and mixed 4:1 for the first two inoculations and 9:1 for the remaining inoculations, with Aluminum Hydroxide (Alum) adjuvant (Brenntag Biosector, Denmark) or with the Ribi-like Sigma Adjuvant system (each vial containing 0.5 mg Monophosphoryl Lipid A from *Salmonella Minnesota* and 0.5 mg synthetic Trehalose Dicorynomycolate in 2% oil [squalene]-Tween® 80-water) (Sigma-Aldrich, St. Louis, MO). Animals were immunized as shown in Table 1 and Supplementary Table 1. Mice received bi-weekly subcutaneous inoculations of 50 μg of the p13Bri and subsequent inoculations were reduced to 20 μg of immunogen. Bleedings were done 7 days after each inoculation starting after the second injection. Differential antibody titers to Aβ1–40 and 1–42 were determined by enzyme-linked immunosorbent analysis (ELISA); the plasma for any bleeding was diluted 1:150 with 50 mM Tris-Saline pH 7.2, 0.1% Tween 20 (TBS-T) and incubated on Immulon 2HB 96-well (Thermo, Waltham, MA) microtiter ELISA plates pre-coated with either 50 ng/well of Aβ1–40 or Aβ1–42 in 50 mM ammonium bicarbonate solution pH 9.6 preincubated at RT for 6 and 24–48 hs respectively, as previously described^21^. Bound antibodies were detected with horseradish peroxidase-labeled goat anti-mouse IgG (H + L) (GE Healthcare UK) or goat anti-mouse IgM(µ) (KPL Gaithersburg, MD, USA). The color developing substrate was Tetramethyl benzidine (TMB) (Pierce, Rockford, IL) and the readings were made at 450 nm. After the 7^th^ inoculation, the M4 mouse was rested for 45 days before being injected intravenously (i.v.) with 10 μg of p13Bri without adjuvant; the terminal bleeding was performed 4 days later, before harvesting the spleen for fusion. The remaining animals were inoculated s.c. 5 more times, for a total of twelve inoculations, with 20 μg of p13Bri, rested for two and a half months at which time an i.v. injection of 10 μg of p13Bri with no adjuvant was given to all the animals. Terminal bleedings and spleen harvesting for fusion were performed 4 days later. The spleens of M1 and M2 and the spleens of M3 and M5 were combined for only two fusions.

### Monoclonal Production

Mouse M4 was sacrificed 165 days after the first inoculation. The spleen was taken and splenocytes were gently dislodged and fused to SP2/0-IL6 cells (ATCC® CRL-2016™) using Polyethylene Glycol 1500 (Sigma-Aldrich St. Louis, MO). Fusion mixture was recovered overnight at 3 million pre-fusion viable cells per ml. Half of the fusion was cryopreserved and from the other half cells were plated the following day in a 96-well plate at 75,000 pre-fusion viable nucleated splenocytes per well at a final volume of 200 μl/well. Cells were cultured for 7 days, then the cells were fed and after 3 days screenings began. The media used was Gibco® Hybridoma-SFM (Fisher Scientific, USA); 15% Fetal Bovine Serum, Hybridoma Fusion and Cloning supplement (HFCS) (Sigma-Aldrich, St. Louis, MO) −2x for the fusion and selection in HAT and 1x during the screening-; 1x Gibco® HT Supplement (Fisher Scientific, Waltham, MA), 10 μg Gentamicin sulfate/ml (Fisher Scientific, Waltham, MA).

The cloning protocol was a serial dilution done in a 96-well plate, and screening of wells with only one colony. To assess for the presence of possible conformational antibodies in any step of the screening and cloning, approximately 125 μL of cell supernatants were rapidly transferred on ice to our laboratory at NYU Langone Medical Center, diluted 1:1 with TBS-T and 50 µL/well applied to Immulon 2HB 96-well (Thermo, Waltham, MA) microtiter ELISA plates pre-coated with either Aβ1–40, Aβ1–42, PrP^Res^ or purified human paired helical filaments (PHF) from an AD subject, in a 50 mM ammonium bicarbonate solution pH 9.6 as previously described^21, 22^. Bound antibodies were detected with horseradish peroxidase-labeled goat anti-mouse IgG + IgA + IgM(H + L) (KPL, Gaithersburg, MD, USA). The color developing substrate was Tetramethyl benzidine (TMB) (Pierce, Rockford, IL) and the readings were made at 450 nm. Samples that were positive for more than one antigen (positivity being defined as a titer more than three times over the background) were cloned again using the same procedure, followed by testing after three days growth. All clones positive for more than one antigen three times were cultured in 5 ml tubes until saturation. The tubes were centrifuged at 3,000 xg for 10 minutes at 4 °C; supernatants were kept and the pelleted cells were divided into at least four vials containing 2 × 10^6^ cells in 0.5 mL of media diluted in half in DMSO, and cryopreserved in liquid nitrogen for storage and future expansion.

Mice M1, 2, 3 and 5 were sacrificed 332 days after the first inoculation. The splenocytes of mice M1 and M2 and the splenocytes of mice M3 and M5 were combined in equal ratios for two fusions as above described and cryopreserved for future use.

### Partial Purification of Monoclonal Antibodies

Monoclonal antibodies present in the supernatants obtained after the fusion of the splenocytes of M4 CD-1 mouse and partner cells SP2/0-IL6, with cloning by serial dilutions, were partially purified by precipitation with Saturated Ammonium Sulfate (SAS) −761.5 g/Lt at 21 °C^54^. Samples were made 40% in the SAS, incubated at RT for at least 4 hours, centrifuged at 14,000 xg for 15 minutes, the supernatant separated and the precipitate washed with a comparable volume of 40% SAS, centrifuged again and the supernatant pooled with the initial supernatant. The precipitate was fractionated and kept at 4 °C until further use. To assess the partial purification of the monoclonals and the specific reactivity, aliquots were dissolved directly into tricine sample buffer (BioRad, Hercules CA) and electrophoresed at ~1–2 μg/lane. The samples for antibody activity were dissolved in distilled deionized water (DDW) to half the original volume and subsequently brought to the desired dilution with the appropriate buffers for the technique.

### Oligomerization of Neurodegenerative Antigens

Human tissue related studies were performed under a protocol approved by the Institutional Review Board at New York University School of Medicine. In all cases, written informed consent for research was obtained from the patient or legal guardian, and the material used had appropriate ethical approval for use in this project. All patients’ data and samples were coded and handled according to NIH guidelines to protect patients’ identities.

Antigens known to be relevant in different neurodegenerative diseases, i.e. Aβ1–40 and Aβ1–42 (amyloidogenic in Alzheimer’s Disease [AD] and other dementia), α-synuclein (Parkinson disease [PD] and Lewy Body Dementia), prion protein (PrP^Res^) (in prion disease) were polymerized to stable oligomeric states by the same glutaraldehyde methodology used to produce the p13Bri^21, 22^. To produce stable fibrils 1mg/ml of either synthetic Aβ1–40, Aβ1–42 and α-synuclein peptides were incubated in PBS pH 7.2 at 37 °C for at least 72 hours, until most of the peptide produced fibrils as determined by EM. Recombinant deer PrP (kindly provided by Dr. D. Brown, Bath University, UK) was incubated in 50 mM Tris buffer pH 7.4 to obtain aggregated species. Oligomeric/aggregated tau was obtained by purifying PHF from known cases of human Alzheimer’s disease, who fulfilled the National Institute of Aging-Reagan criteria for AD, obtained from the Alzheimer Brain Bank of the Alzheimer’s Disease Center at NYU, as previously described^21, 55^. Briefly, 30 gm of frontal cortex was homogenized in 75 ml of 50 mM Tris-buffered saline (TBS), pH 7.4 using an Ultra Turrox T25 tissue homogenizer (IKA Works, Inc; Staufen, Germany). 75 ml of 20% sarcosyl in H_2_O was added to the sample and it was homogenized again. The homogenized material was centrifuged at 3,500 rpm in a Beckman GPR centrifuge and 6 ml aliquots of the supernatant were each layered over 1 ml TBS/0.1% sulfobetaine 3–14 (SB3–14) (Sigma-Aldrich, St Louis, MO) and centrifuged in an Optima Max ultracentrifuge at 75,000 rpm for 2 hours at 20 °C. Each pellet was resuspended by sonication in 1 ml of 10%NaCl in TBS/0.1%SB3–14 followed by the addition of 6 ml of 10% NaCl in TBS/0.1%SB3–14, layered over 1 ml of 20% sucrose in 10%NaCl TBS/0.1%SB3–14 and centrifuged at 75,000 rpm for 1.5 hours at 20 °C. The final pellets were resuspended in TBS by sonication prior to use. Purified PHF was also treated with proteinase K (Sigma-Aldrich, USA) to release oligomers from fibrils, at 1:100 in PBS pH 7.2 for 30 minutes at 37 °C, immediately quenched with phenylmethanesuphonyl fluoride (PMSF) and either immediately dissolved in sample buffer for use in blots or frozen at −80 °C for future use.

#### Electron Microscopy

Electron microscopy images, using negative staining, to assess the conformational states of monomeric, oligomeric or fibrillar forms of the aggregated and polymerized ABri, p13Bri, Aβ1–40 and Aβ1–42, α-synuclein, PHF and PrP were done as previously described and were taken at the NYULMC OCS Microscopy Core^22^. Samples were diluted 1 mg/ml in PBS pH 7.4 and vortexed before 3 μl of each one were placed onto carbon coated 400 mesh Cu/Rh grid (Ted Pella Inc., Redding, CA). Negative staining was performed using 1% uranyl acetate diluted in distilled water (Polysciences, Inc, Warrington, PA). Stained grids were examined under Philips CM-12 electron microscope and photographed with a Gatan (4k x2.7k) digital camera. In electron micrographs the pixel size is ~3.3 Å/pixel and the defocus is −0.7µm or −768nm. In most cases samples were kept for weeks or months to repeat the EMs at different times to follow the fibrillization or the stability of the oligomers.

#### Immunohistochemistry

Histology was performed on aged (>16 months old) 3xTg^56^ mouse brain sections with extensive Aβ and tau pathology; or formalin fixed paraffin embedded brain cortex sections of human AD, human age-matched controls and human young controls obtained from the Alzheimer Brain Bank of the Alzheimer’s Disease Center at NYU. 40 μm mouse brains sections fixed with periodate-lysine-paraformaldehyde (PLP), kept in DMSO cryoprotectant, were washed three times for 5 minutes with PBS pH 7.2 and twice for 15 min with 0.3% hydrogen peroxide to quench endogenous peroxidase activity. Sections were then blocked with MOM kit Blocking solution (Vector Laboratories, Burlingame, CA) following the manufacture’s protocol and incubated overnight with conformational hyperimmune 3xTg or CD-1 (M4) mouse plasma diluted 1:300 in MOM kit Diluent Solution. The following day, sections were washed three times with PBS and incubated 1 hour with biotinylated anti-mouse IgG (H + L) or anti-mouse IgM (µ specific chain) antibodies (Vector Laboratories, Burlingame, CA) diluted in PBS 1:1000 followed by 1 hour incubation of Vectastain® AB solution (Vector Laboratories, Burlingame, CA) as indicated by the manufacturer’s protocol. Slides were developed with 3,3-diaminobenzidine tetrahydrochloride with 2.5% nickel ammonium sulfate (Acros Organics, NJ) diluted in 0.2 M sodium acetate (NaAc) pH 6. The reaction was stopped by removal of the nickel solution and extensively rinsing with 0.2 M NaAc before stabilizing with PBS and further mounting on glass slides with Depex® Mounting Media (Electron Microscopy Sciences, Hatfield, PA).

Paraffin embedded human brain sections were dewaxed and rehydrated with successive washes of xylene (2 × 5 minutes), 100% ethanol (2 × 5 minutes), 95% ethanol (5 minutes), 70% ethanol (5 minutes) and PBS (5 minutes). Next, slides underwent antigen retrieval by boiling for 20 minutes in 10mM sodium citrate-0.05%-Tween20 pH 6.0. Sections were then washed with PBS (3x 5 minutes), followed by 0.3% hydrogen peroxide washing, twice 15 minutes each. Next slides were washed with PBS (3x 5 minutes) and blocked 1 hour at RT with 10% normal goat serum [NGS] (Thermo Scientific)−0.2% Triton X-100 (Sigma-Aldrich, St Louis, MO) in PBS. Slides were incubated overnight with the plasmas of hyperimmune 3xTg or CD-1 (M4) mice diluted 1:300 in 3% NGS-0.2% Triton X-100. Slides were then washed three times with PBS and incubated for 1 hour with biotinylated anti-mouse IgM (µ specific chain) antibody diluted 1:1000 in PBS followed by 1 hour incubation of Vectastain® AB solution. Slides were further developed with 3,3-diaminobenzidine tetrahydrochloride with nickel ammonium sulfate as described above.

To assess the reactivity of the monoclonal antibodies obtained after the fusion, Human brain sections were treated as above described but no antigen retrieval was performed. Monoclonal antibodies 23B, 12E, 10E and 10F were used with a dilution of 1:2500 and monoclonal antibody 3D was used at 1:3000.

For immunofluorescence, paraffin embedded human AD brain slides were dewaxed and rehydrated as described above, then blocked for 1 hour at RT with 10% NGS-0.2% Triton X-100 in PBS and then incubated overnight at 4 °C with plasma of a CD-1 (M4) mouse hyperimmunized with p13pBri, diluted 1:300 in PBS-T. Slides were then washed with PBS (3x 5 minutes) and incubated 2 hours with Alexa fluor® 488-conjugated goat anti-mouse IgM and Alexa fluor® 647-conjugated goat anti-mouse IgG (Jackson ImmunoResearch, West Groove, PA) both diluted 1:500 in PBS, followed by 10 minutes incubation with bisBenzimide H 33342 trihydrochloride (Sigma-Aldrich, St. Louis, MO) diluted 1 μl/ml. Slides were then washed in PBS (3x 5 minutes) and coverslipped with PermaFluor™ Aqueous Mounting Medium (Thermo Scientific, Waltham, MA).

All slides were first screened on a Leica DM LB 100T microscope, than scanned using a Hamamatsu Nanozoomer 2.0HT Digital Slide Scanner (Hamamatsu, Shizuoka Prefecture, Japan) at the NYU OCS Experimental Pathology Histology Core. The images were viewed using the Slidepath software (Leica, Wetzlar, Germany).

#### Electrophoresis and Western blot

To characterize the monoclonal antibodies obtained after the fusion and cloning, 1 μg of each antibody were mixed with an equal volume of tricine sample buffer, electrophoresed on Bolt™ 4–12% Bis-Tris gels and buffer (Thermo, Waltham, MA) under non-reducing conditions and transferred onto nitrocellulose membranes for 1 hour at 386mA in 0.1% 3-(Cyclohexylamino)-1-propanesulfonic acid (CAPS) (Sigma-Aldrich, St. Louis, MO)-10% methanol. To assess equal protein loading in each lane, membranes were stained for 1 min with reversible 0.1% Fast Green FCF (Sigma-Aldrich CO, USA), as per the manufacturer’s instructions, in 25% methanol-10% Acetic Acid. The background was de-stained with rapid changes of 25% methanol, followed by transfer to distilled water before scanning on a Canon F916900 scanner Canon Inc, China). Membranes were then washed in TBS-T for at least 15 minutes (until the reversible stain was removed from the proteins on the membrane), blocked with 5% non-fat dry milk in TBS-T pH 8.3, for 1 h at RT, washed three times with TBS-T, and then incubated 45 minutes with anti-mouse μ chain specific diluted 1:8000 (KPL, Gaithersburg, MD) or anti-mouse Kappa diluted 1:5000 (Southern Biotech, Birmingham, AL). Bound antibodies were detected with the ECL detection system (Pierce, Rockford, IL) on autoradiography films (MIDSCI, St Louis, MO).

To determine the reactivity of the anti-conformational monoclonal antibodies to Aβ1–40, Aβ1–42, α-synuclein, PHF, PrP^Res^, 22L, sheep scrapie and deer PrP, each peptide or protein was loaded 1–2 μg/lane and electrophoresed on Bolt™ 4–12% Bis-Tris gels (Thermo, Waltham, MA) under non-reducing conditions using 3 μl of High range Rainbow™ molecular weight marker (Amersham Biosciences, Piscataway, NJ) and later transferred onto nitrocellulose membranes for 1 hour at 386 mA in 0.1% CAPS-10% methanol. To assess the protein loading in each lane, the membranes were first stained with reversible 0.1% Fast Green FCF as described above. Membranes were scanned before being blocked with 5% non-fat dry milk in TBS-T pH 8.3, for 1 h at RT and washed three times with TBS-T. Membranes were then incubated with each monoclonal antibody, diluted 1:750 in TBS-T, or monoclonal anti-Aβ antibodies 4G8/6E10 (1:4000) (BioLegend, San Diego, CA), monoclonal anti-α-synuclein Ab-2 (1:3000) (Thermo, Waltham, MA), PHF-1 (1:2000) (which recognizes phosphorylated serine in positions 396 and 404, kindly provided by Dr. Peter Davies from the Feinstein Institute for Medical Research, Manhasset, NY) or anti-PrP antibodies 7D9/6D11 (1:8000) (BioLegend, San Diego, CA). Membranes were incubated later with peroxidase-linked anti-mouse IgG (GE Healthcare UK) (1:4000) for anti-α-synuclein, PHF-1 and 7D9/6D11. To detect bound monoclonal antibodies anti-mouse μ chain specific was used diluted at 1:8000 (KPL, Gaithersburg, MD).

## Electronic supplementary material

Supplementary Figures and Table

## References

[1] Viola KL, Klein WL (2015). Amyloid beta oligomers in Alzheimer’s disease pathogenesis, treatment, and diagnosis. Acta Neuropathol.

[2] Jucker M, Walker LC (2011). Pathogenic protein seeding in alzheimer disease and other neurodegenerative disorders. Ann. Neurol.

[3] Riek R, Eisenberg DS (2016). The activities of amyloids from a structural perspective. Nature.

[4] Scheltens P (2016). Alzheimer’s disease. Lancet.

[5] Nelson PT (2012). Correlation of Alzheimer’s disease neuropathologic changes with cognitive status: a review of the literature. JNEN.

[6] Tomic JL, Pensalfini A, Head E, Glabe CG (2009). Soluble fibrillar oligomer levels are elevated in Alzheimer’s disease brain and correlate with cognitive dysfunction. Neurobiol Dis.

[7] Ashe KH, Aguzzi A (2013). Prions, prionoids and pathogenic proteins in Alzheimer disease. Prion.

[8] Knight EM (2016). Effective anti-Alzheimer Abeta therapy involves depletion of specific Abeta oligomer subtypes. Neurol Neuroimmunol Neuroinflamm.

[9] Selkoe DJ, Hardy J (2016). The amyloid hypothesis of Alzheimer’s disease at 25 years. EMBO Mol Med.

[10] Walker LC, Diamond MI, Duff KE, Hyman BT (2013). Mechanisms of protein seeding in neurodegenerative diseases. JAMA Neurol.

[11] Prusiner SB (2012). Cell biology. A unifying role for prions in neurodegenerative diseases. Science.

[12] Cobb NJ, Apostol MI, Chen S, Smirnovas V, Surewicz WK (2014). Conformational stability of mammalian prion protein amyloid fibrils is dictated by a packing polymorphism within the core region. J Biol Chem.

[13] Mao, X. *et al*. Pathological alpha-synuclein transmission initiated by binding lymphocyte-activation gene 3. *Science* **353** (2016). 10.1126/science.aah3374 PMC5510615 27708076

[14] Wisniewski T, Goni F (2015). Immunotherapeutic Approaches for Alzheimer’s Disease. Neuron.

[15] Wisniewski T, Drummond E (2016). Developing Therapeutic Vaccines Against Alzheimer’s Disease. Expert Rev Vaccines.

[16] Orgogozo JM (2003). Subacute meningoencephalitis in a subset of patients with AD after A beta 42 immunization. Neurology.

[17] Crespi GA, Ascher DB, Parker MW, Miles LA (2014). Crystallization and preliminary X-ray diffraction analysis of the Fab portion of the Alzheimer’s disease immunotherapy candidate bapineuzumab complexed with amyloid-beta. Acta Crystallogr F Struct Biol Commun.

[18] Kayed R, Glabe CG (2006). Conformation-dependent anti-amyloid oligomer antibodies. Methods Enzymol.

[19] Vidal R (1999). A stop-codon mutation in the BRI gene associated with familial British dementia. Nature.

[20] Rostagno A (2005). Chromosome 13 dementias. Cell Mol. Life Sci.

[21] Goni F (2010). Immunomodulation targeting abnormal protein conformation reduces pathology in a mouse model of Alzheimer’s disease. PLoS. ONE.

[22] Goni F (2013). Immunomodulation targeting both Ab and tau pathological conformers ameliorates Alzheimer’s Disease pathology in TgSwDI and 3xTg mouse models. Journal of Neuroinflammation.

[23] Moore BD, Rangachari V, Tay WM, Milkovic NM, Rosenberry TL (2009). Biophysical analyses of synthetic amyloid-beta(1-42) aggregates before and after covalent cross-linking. Implications for deducing the structure of endogenous amyloid-beta oligomers. Biochemistry.

[24] Migneault, I., Dartiguenave, C., Bertrand, M. J. & Waldron, K. C. Glutaraldehyde: behavior in aqueous solution, reaction with proteins, and application to enzyme crosslinking. *Biotechniques* **37**, 790–796, 798–802 (2004). 10.2144/04375RV01 15560135

[25] Sawaya MR (2007). Atomic structures of amyloid cross-beta spines reveal varied steric zippers. Nature.

[26] Avila J (2016). Tau Structures. Front Aging Neurosci.

[27] Abskharon RN (2012). A novel expression system for production of soluble prion proteins in E. coli. Microb Cell Fact.

[28] Ostapchenko VG (2010). Two amyloid States of the prion protein display significantly different folding patterns. J Mol Biol.

[29] Lauren J, Gimbel DA, Nygaard HB, Gilbert JW, Strittmatter SM (2009). Cellular prion protein mediates impairment of synaptic plasticity by amyloid-beta oligomers. Nature.

[30] Wiltzius JJ (2009). Molecular mechanisms for protein-encoded inheritance. Nat Struct Mol Biol.

[31] Wisniewski T, Goni F (2014). Immunotherapy for Alzheimer’s disease. Biochemical Pharmacology.

[32] Sigurdsson EM (2004). An attenuated immune response is sufficient to enhance cognition in an Alzheimer’s disease mouse model immunized with amyloid-b derivatives. J. Neurosci.

[33] Barrow CJ, Yasuda A, Kenny PT, Zagorski MG (1992). Solution conformations and aggregational properties of synthetic amyloid beta-peptides of Alzheimer’s disease. Analysis of circular dichroism spectra. J. Mol. Biol.

[34] Daebel V (2012). beta-Sheet core of tau paired helical filaments revealed by solid-state NMR. J Am Chem Soc.

[35] von Bergen M (2000). Assembly of tau protein into Alzheimer paired helical filaments depends on a local sequence motif ((306)VQIVYK(311)) forming beta structure. Proc Natl Acad Sci USA.

[36] Kayed R (2003). Common structure of soluble amyloid oligomers implies common mechanism of pathogenesis. Science.

[37] Lee EB (2006). Targeting amyloid-beta peptide (Abeta) oligomers by passive immunization with a conformation-selective monoclonal antibody improves learning and memory in Abeta precursor protein (APP) transgenic mice. J Biol. Chem.

[38] Tucker S (2015). The murine version of BAN2401 (mAb158) selectively reduces amyloid-beta protofibrils in brain and cerebrospinal fluid of tg-ArcSwe mice. J Alzheimers Dis.

[39] Lambert MP (2007). Monoclonal antibodies that target pathological assemblies of Abeta. J Neurochem.

[40] Hillen H (2010). Generation and therapeutic efficacy of highly oligomer-specific beta-amyloid antibodies. J Neurosci.

[41] Rasool S, Martinez-Coria H, Wu JW, LaFerla F, Glabe CG (2013). Systemic vaccination with anti-oligomeric monoclonal antibodies improves cognitive function by reducing Abeta deposition and tau pathology in 3xTg-AD mice. J. Neurochem.

[42] Dorostkar MM (2014). Immunotherapy alleviates amyloid-associated synaptic pathology in an Alzheimer’s disease mouse model. Brain.

[43] Castillo-Carranza DL (2015). Tau immunotherapy modulates both pathological tau and upstream amyloid pathology in an Alzheimer’s disease mouse model. J Neurosci.

[44] O'Nuallain B, Wetzel R (2002). Conformational Abs recognizing a generic amyloid fibril epitope. Proc Natl Acad Sci USA.

[45] Goure WF, Krafft GA, Jerecic J, Hefti F (2014). Targeting the proper amyloid-beta neuronal toxins: a path forward for Alzheimer’s disease immunotherapeutics. Alzheimers Res Ther.

[46] Habicht G (2007). Directed selection of a conformational antibody domain that prevents mature amyloid fibril formation by stabilizing Abeta protofibrils. Proc Natl Acad Sci USA.

[47] Kayed R (2007). Fibril specific, conformation dependent antibodies recognize a generic epitope common to amyloid fibrils and fibrillar oligomers that is absent in prefibrillar oligomers. Mol. Neurodegener.

[48] Rasool S (2012). Vaccination with a non-human random sequence amyloid oligomer mimic results in improved cognitive function and reduced plaque deposition and micro hemorrhage in Tg2576 mice. Mol. Neurodegener.

[49] Hamilton RL (2000). Lewy bodies in Alzheimer’s disease: a neuropathological review of 145 cases using alpha-synuclein immunohistochemistry. Brain Pathol.

[50] White LR (2016). Neuropathologic comorbidity and cognitive impairment in the Nun and Honolulu-Asia Aging Studies. Neurology.

[51] Schneider JA, Arvanitakis Z, Bang W, Bennett DA (2007). Mixed brain pathologies account for most dementia cases in community-dwelling older persons. Neurology.

[52] James, B. D. *et al*. TDP-43 stage, mixed pathologies, and clinical Alzheimer’s-type dementia. *Brain* (2016). 10.1093/brain/aww224 PMC5091047 27694152

[53] Hara H (2016). An Oral Abeta Vaccine Using a Recombinant Adeno-Associated Virus Vector in Aged Monkeys: Reduction in Plaque Amyloid and Increase in Abeta Oligomers. J Alzheimers Dis.

[54] Berasain P, Carmona C, Frangione B, Dalton JP, Goni F (2000). Fasciola hepatica: parasite-secreted proteinases degrade all human IgG subclasses: determination of the specific cleavage sites and identification of the immunoglobulin fragments produced. Exp Parasitol.

[55] Wrzolek MA (1992). Immune electron microscopic characterization of monoclonal antibodies to Alzheimer neurofibrillary tangles. Am J Pathol.

[56] Oddo S (2003). Triple-transgenic model of Alzheimer’s disease with plaques and tangles: intracellular Abeta and synaptic dysfunction. Neuron.

