# Supplementary material for: Production of Monoclonal Antibodies to Pathologic β-sheet Oligomeric Conformers in Neurodegenerative Diseases
Source: Sci Rep. 2017 Aug 29;7:9881. doi: 10.1038/s41598-017-10393-z (PMC5575137; doi:10.1038/s41598-017-10393-z)
Supplement: Supplementary file 1 — Supplementary Figures and Table [file 41598_2017_10393_MOESM1_ESM.pdf]

Supplemental Information for:

Production of Monoclonal Antibodies to Pathologic β-sheet Oligomeric Conformers in Neurodegenerative Diseases

Fernando Goñi[1*], Mitchell Martá-Ariza[1], Daniel Peyser[1], Krystal Herline[1] and Thomas Wisniewski[1,2,3*]

[1]Center for Cognitive Neurology and Department of Neurology, New York University School of Medicine, New York, New York, USA. Departments of [2]Pathology and [3]Psychiatry, New York University School of Medicine, New York, New York, USA.

*Correspondence should be addressed to T.W. (Thomas.Wisniewski@nyumc.org) or F.G. (Fernando.Goni@nyumc.org)

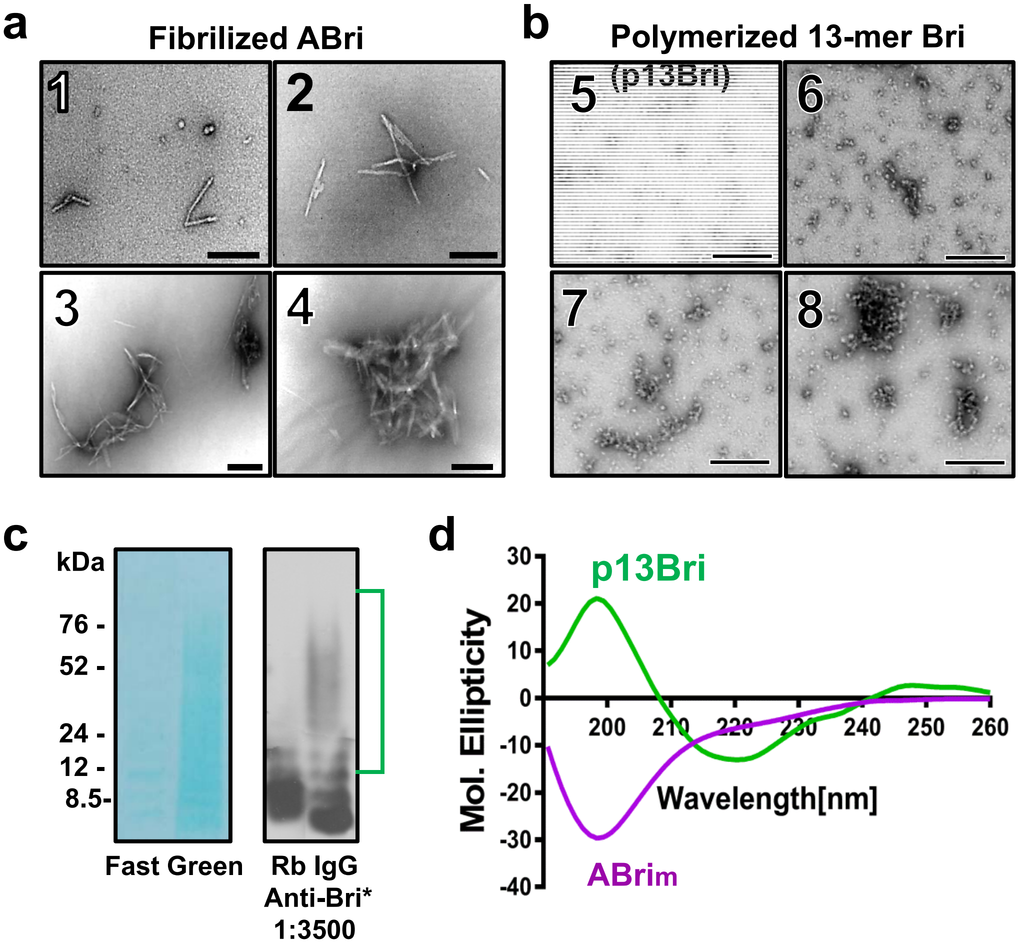

Supplementary Figure 1. Characterization of the ABri and Polymerized immunogenic 13-mer Bri (p13Bri) Peptides. a) Electron microscopy (EM) of the sequential fibrillization of the ABri peptide; 1) 24 hours incubation at room temperature (RT); 2) one week incubation at RT with associated fibrils; 3) and 4) two weeks incubation at RT, big clusters of precipitated long fibrils. Scale bars represent 200 µm. b) EM images of 13-mer Bri peptide after controlled polymerization with glutaraldehyde (p13Bri) showing different oligomeric states; 5) two hours after preparation of the sample; 6) one week after incubation at RT and, 7) and 8) after two weeks of incubation at RT. Scale bars represent 200 µm. c) Nitrocellulose blot from 15% SDS-PAGE of the ABri (lane 1) and p13Bri (lane 2) peptides. Left panel, fast green protein reversible stain; right panel immunoblot with anti-Bri antibody kindly provided by Dr. R. Vidal IUPUI. Green bracket marks oligomeric state between 10-100 kDa. d) Circular dichroism of the p13Bri peptide all β structure (green) and ABri monomer (purple).

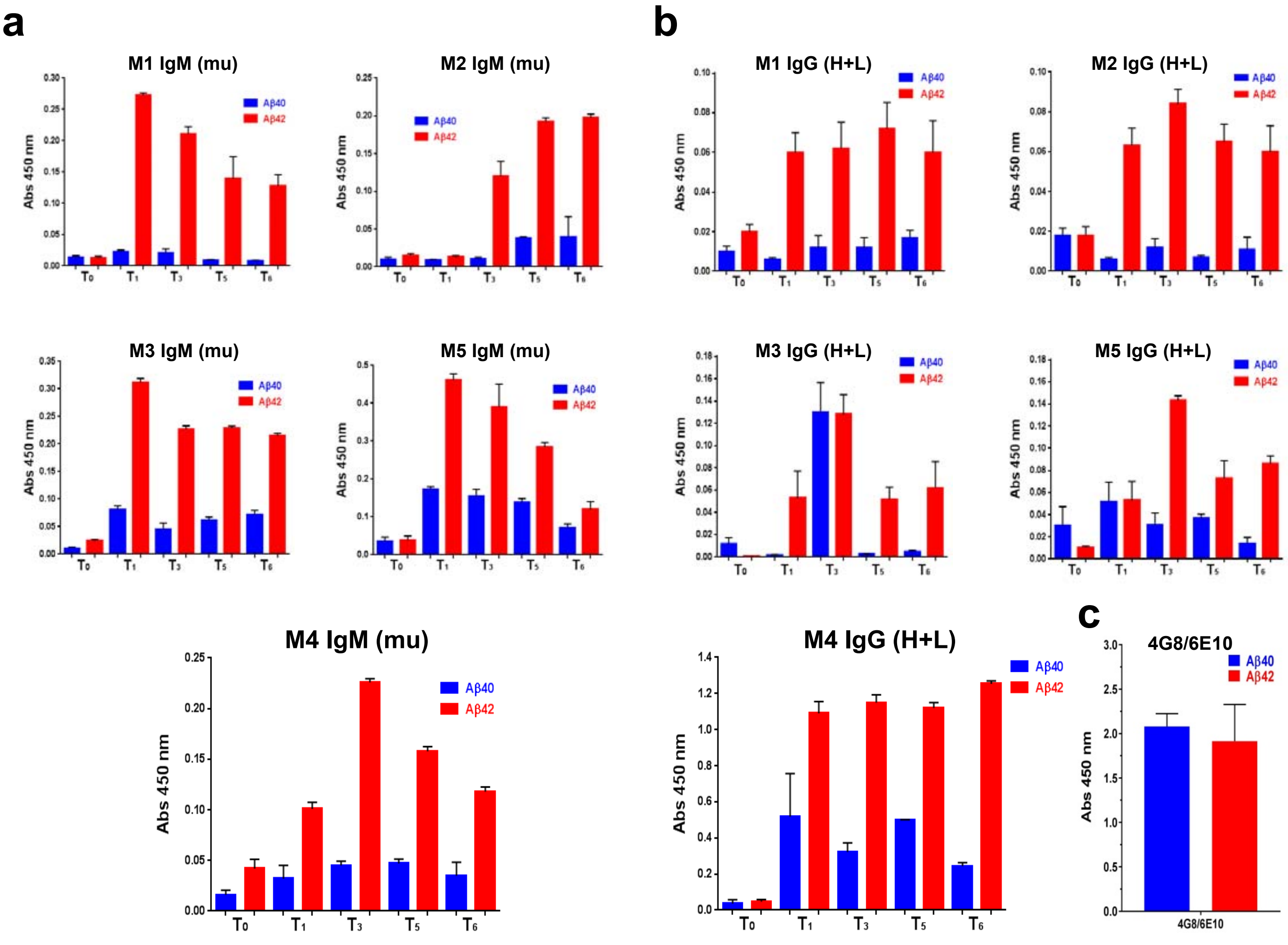

Supplementary Figure 2. Plasma Levels of Anti-Aβ 1-40 and Anti-Aβ 1-42 from p13Bri Inoculated CD-1 Mice. a) ELISA data showing plasma reactivity to Aβ40 and Aβ42 from different bleedings of all CD-1 mice as per supplementary table 1, using peroxidase-labelled goat anti-mouse IgM (μ chain). Samples were run on triplicate. b) ELISA data showing plasma reactivity to Aβ40 and Aβ42 from different bleedings of all CD-1 mice using peroxidase-labelled goat anti-mouse IgG (H+L). Samples were run on triplicate. c) Plate coating control showing similar reactivity detected for Aβ40 and Aβ42 with commercial antibodies 4G8/6E10 and secondary anti-mouse IgG (H+L).

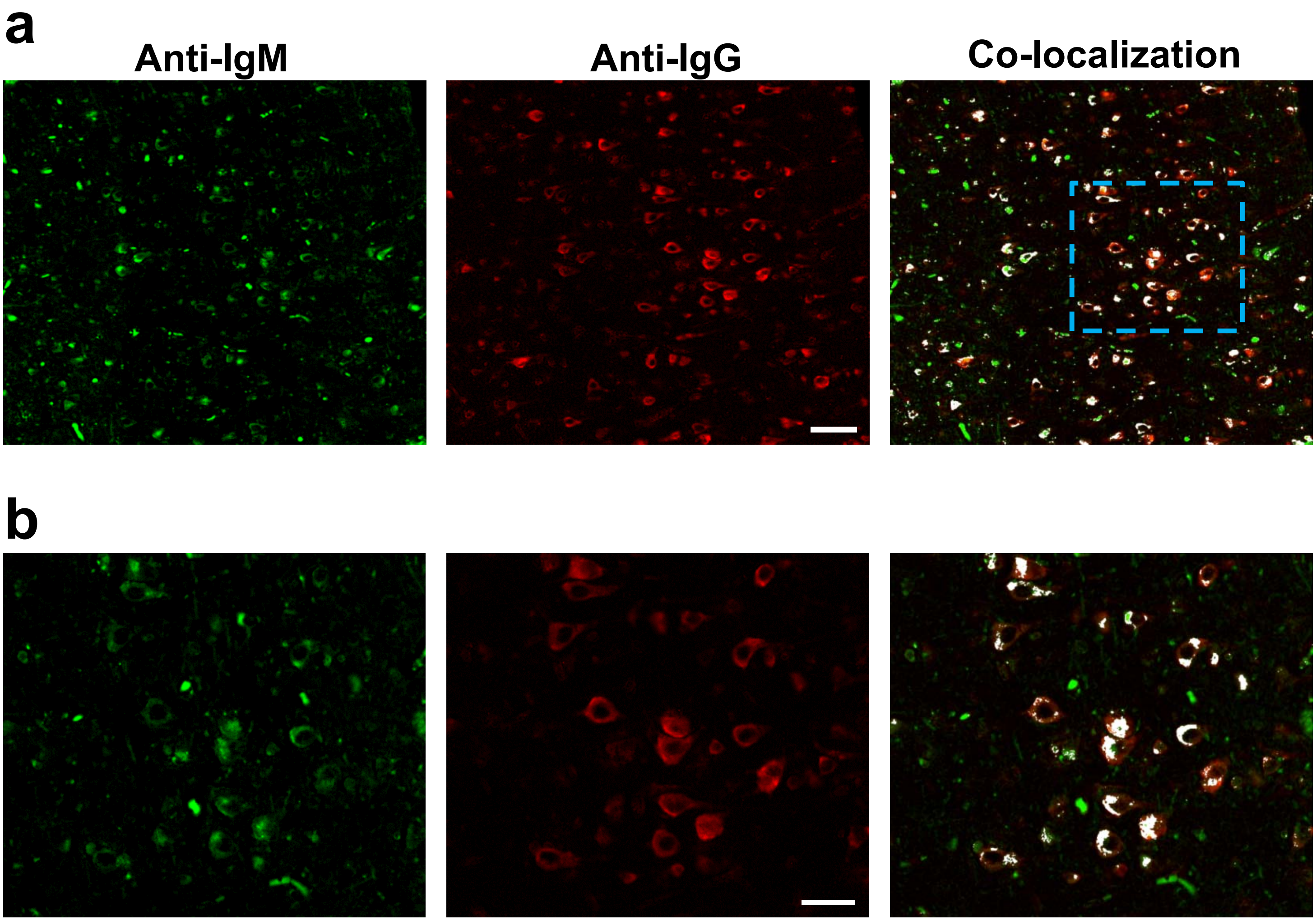

Supplementary Figure 3. Co-localization on Human AD brains of IgM and IgG antibodies present in the plasma of the p13Bri inoculated CD-1 M4 mouse: a) Representative images showing the co-localization on the cortex of human AD brains of IgM and IgG antibodies present in the plasma of CD-1 M4 mouse inoculated with p13Bri. A combined T6+Tf pool was used as per table 1. b) Higher magnification of the boxed area in a).

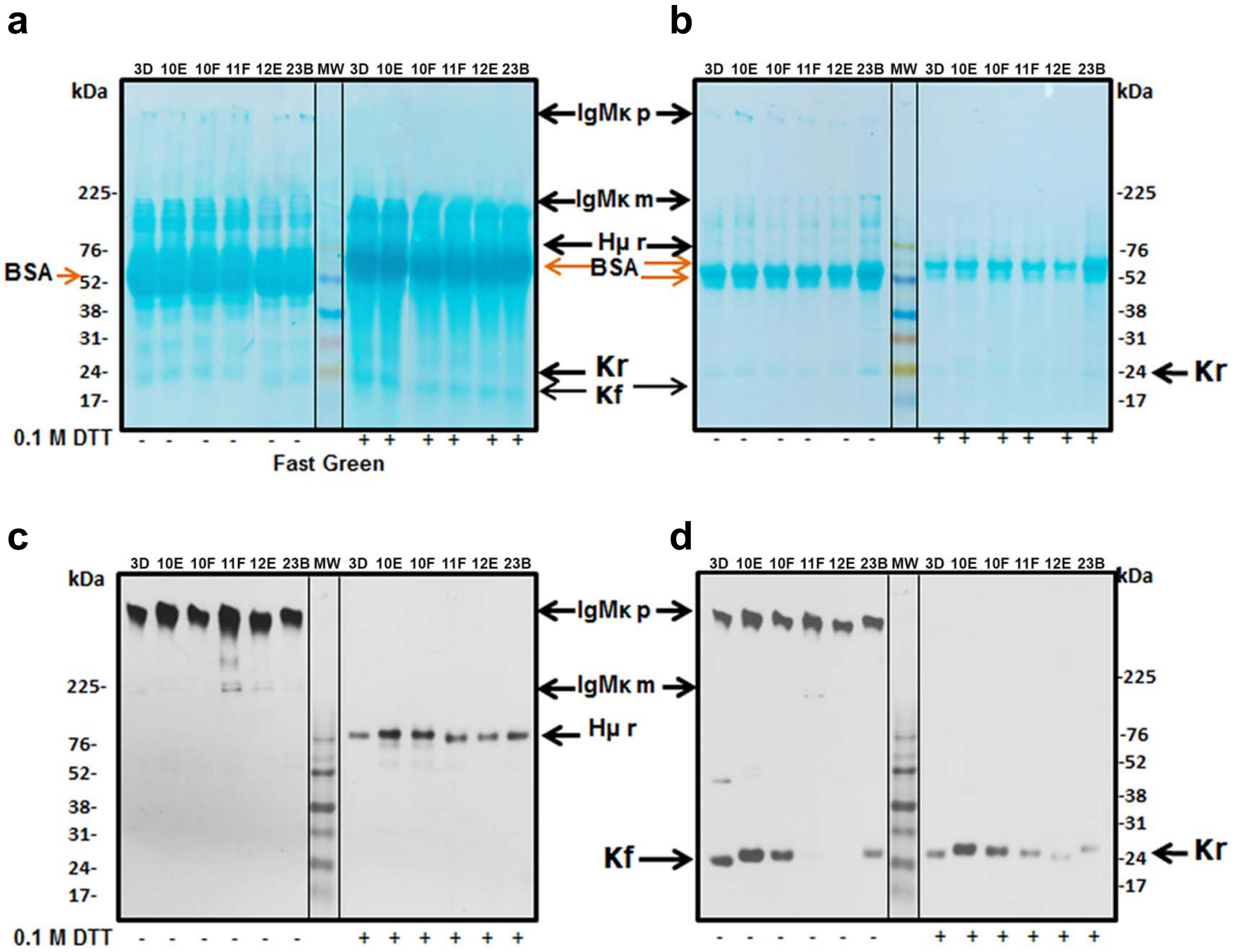

Supplementary Figure 4. Purification and Characterization of anti β-sheet secondary structure conformational monoclonal antibodies obtained from the fusion of p13Bri hyper-immunized M4 CD-1 mouse spleen cells with SP2/mIL-6 fusion partner. a) Fast Green of cell supernatants of hybridomas 3D, 10E, 10F, 11F, 12E and 23B obtained from the fusion of spleen cells of p13Bri immunized M4 mouse and SP2/0-IL6 fusion partner. Large amount of bovine serum albumin (BSA) from the growth media supplementation is shown. b-d). Western blot of the 40% saturated Ammonium Sulfate (SAS) purified antibodies from clones 3D, 10E, 10F, 11F, 12E and 23B. b) Fast Green stain with residual BSA; c) Anti-mouse IgM μ reactivity and, d) anti-mouse Kappa reactivity. Left part of the blots show untreated samples and right part 0.1M Dithiothreitol (DTT) disulfide bridges reduced samples. IgMk p: pentameric IgM; IgMk m: monomeric IgM; Hμ r: mu Heavy chain reduced; Kf: free kappa Light chains and Kr: reduced kappa light chains.

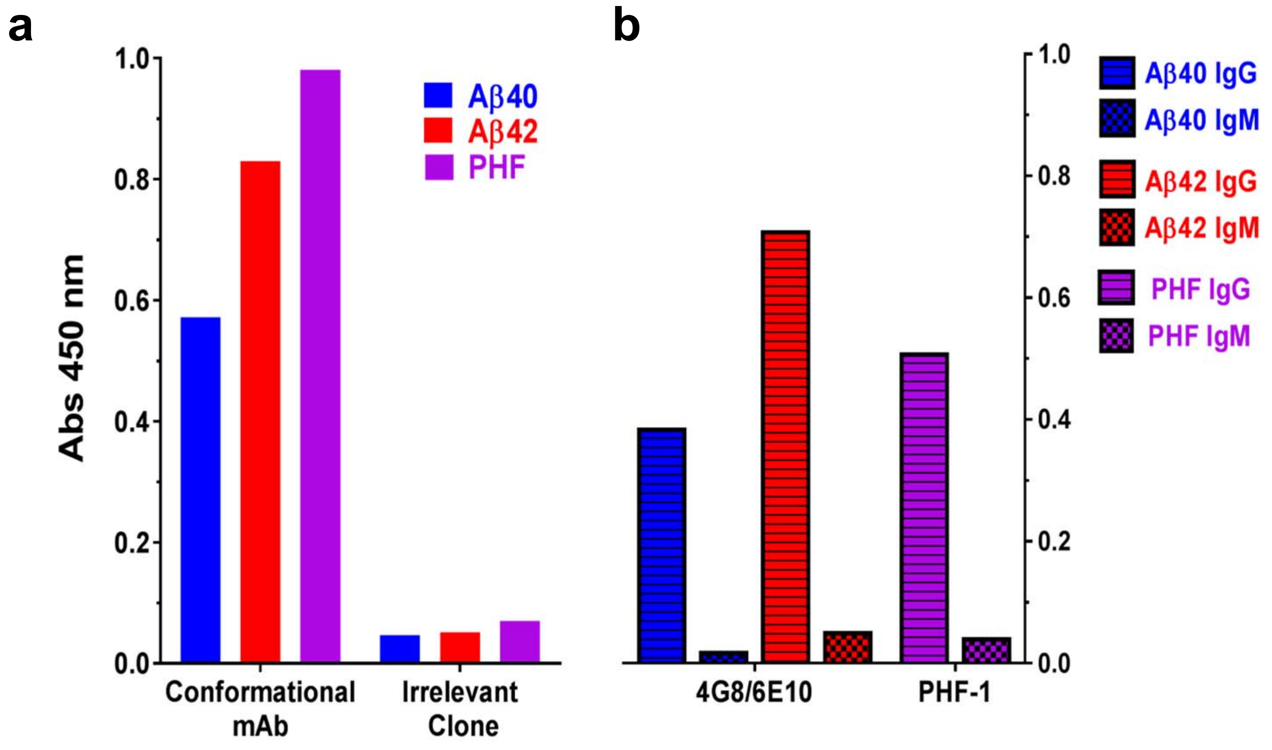

Supplementary Figure 5. Control of protein/peptide coat and IgM reactivity in ELISA plates of Aβ1-40, Aβ1-42 and PHF. a) Typical ELISA plates coated with Aβ1-40, Aβ1-42 or PHF as per Figure 2, showing the difference between a clone with potential conformational monoclonal antibody and an irrelevant clone to mark the background IgM reactivity on a cell supernatant that had comparable number of cells. b) Control of the coat in each ELISA plate with commercial mouse IgG anti-Aβ 4G8 and 6E10 and commercial IgG anti-PHF PHF-1. Background anti-IgM reactivity is similar to the irrelevant clone.

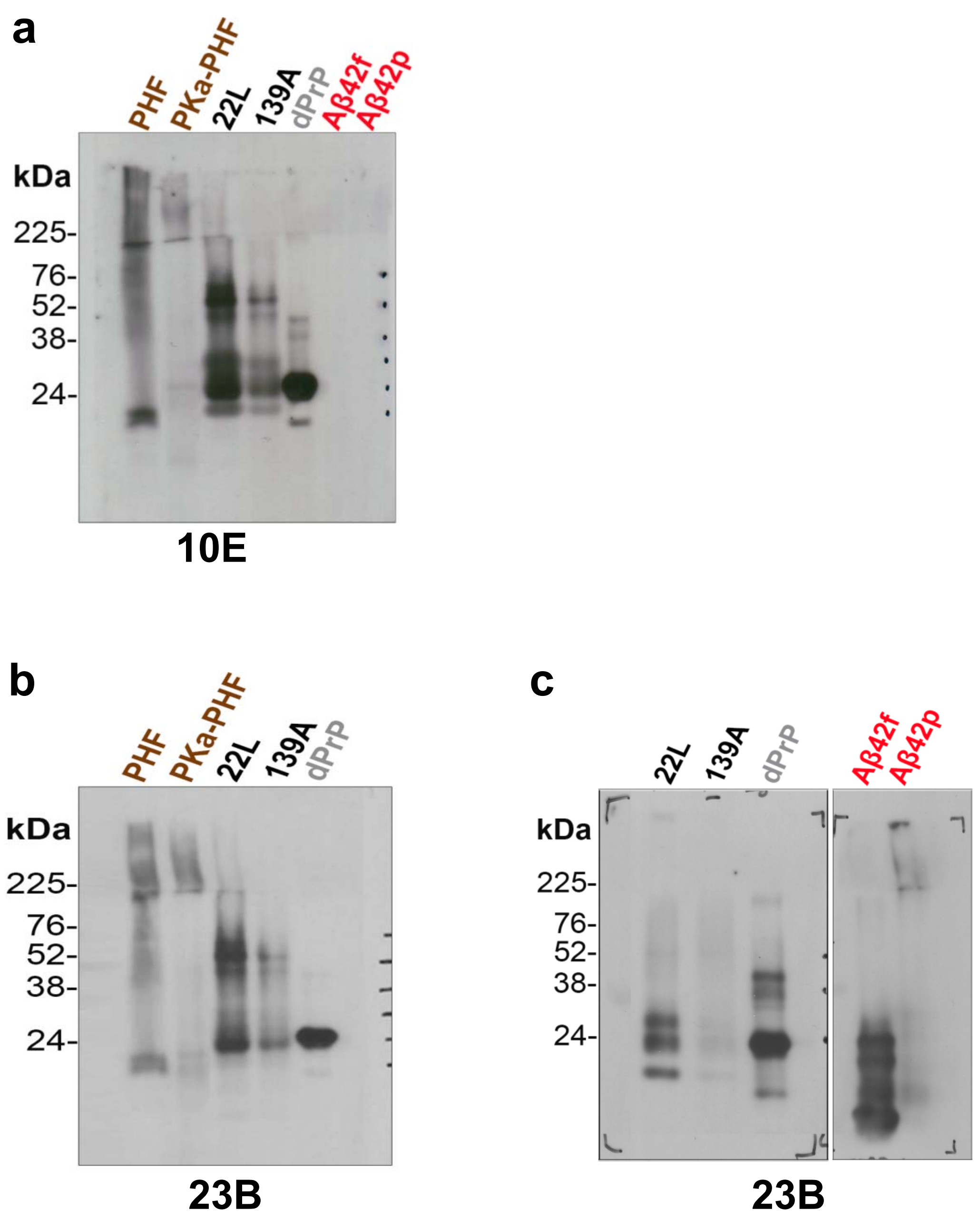

Supplementary Figure 6. Reactivity of anti β-sheet secondary structure conformational monoclonal antibodies 10E and 23B against Paired Helical Filaments (PHF), Amyloid-β 42 and PrP preparations in uncropped blots that are shown in figure 3 of the main manuscript. a. Western blot showing the reactivity of conformational mAb 10E against PHF and PKa digested PHF, scrapie 22L PrP^{Res}, scrapie 139A PrP^{Res} and dPrP. **b.** Conformational mAb 23B reactivity against PHF, Pka digested PHF, scrapie 22L PrP^{Res}, scrapie 139A PrP^{Res} and dPrP. **c.** Reactivity of the conformational mAb 23B against scrapie 22L PrP^{Res}, scrapie 139A PrP^{Res}, dPrP, fibrilized Aβ (Aβ42f) and polymerized Aβ42 (Aβ42p).

Supplementary Table 1. Protocol for the Immunization of CD-1 Mice (M1 to M5) with p13Bri for the Production of anti-conformational Antibodies.

Date of Inoculation (in days)	p13Bri Antigen (µg/animal)*	Antigen to Adjuvant Ratio**	Route of Inoculation	Identification of Bleed	Date of Bleed (in days)
-	---	---	---	Pre-Immune T0	-7
0	50	4:1	s.c.	-	-
14	50	4:1	s.c.	-	-
-	-	-	-	T1	21
28	20	9:1	s.c.	-	-
-	-	-	-	T2	35
49	20	9:1	s.c.	-	-
-	-	-	-	T3	56
69	20	9:1	s.c.	-	-
-	-	-	-	T4	76
91	20	9:1	s.c.		
-	-	-	-	T5	98
119	20	9:1	s.c.		
-	-	-	-	T6	126
140	20 (no M4)	9:1	s.c.		
-	-	-	-	T7	147
161	20 (no M4)	9:1	s.c.		
-	-	-	-	T8	168
165	**10 (only M4)**	**no adjuvant**	**i.v.**	**-**	-
169	**M4 Fusion to SP2/0-IL6*** **	**-**	**-**	**terminal bleeding M4**	169
181	20	9:1	s.c.		
-	-	-	-	T9	189
201	20	9:1	s.c.		
-	-	-	-	T10	208
258	20	9:1	s.c.		
-	-	-	-	T11	265
332	**10 (M1, M2, M3,M5)**	**no adjuvant**	**i.v.**	**-**	-
336	**M1+M2 Fused SP2/0-IL6*** **	**-**	**-**	**TTB M1,M2, M3,M5**	336
336	**M3+M5 Fused SP2/0-IL6*** **	**-**	**-**	**TTB M1,M2, M3,M5**	336
-	**-**	**-**	**-**	**M1+M2 Frozen**	341
-	**-**	**-**	**-**	**M3+M5 Frozen**	341

* All animals inoculated unless indicated. **: Alum M1, M2; Sigma adjuvant system: M3, M4, M5. s.c.: subcutaneous. i.v.: intravenous. TTB: Terminal bleeding. ***: Fusion partner
